# Regionalized differentiation of CRH, TRH, and GHRH peptidergic neurons in the mouse hypothalamus

**DOI:** 10.1007/s00429-013-0554-2

**Published:** 2013-04-30

**Authors:** Nicanor Morales-Delgado, Beatriz Castro-Robles, José L. Ferrán, Margaret Martinez-de-la-Torre, Luis Puelles, Carmen Díaz

**Affiliations:** 1 Department of Medical Sciences, School of Medicine, Regional Centre for Biomedical Research and Institute for Research in Neurological Disabilities, University of Castilla-La Mancha, Calle Almansa, 14, 02006 Albacete, Spain; 2 Department of Human Anatomy and Psychobiology, School of Medicine, University of Murcia, Murcia, Spain

**Keywords:** Forebrain, Hypothalamus, *Crh*, *Trh*, *Ghrh*, Migrations

## Abstract

According to the updated prosomeric model, the hypothalamus is subdivided rostrocaudally into terminal and peduncular parts, and dorsoventrally into alar, basal, and floor longitudinal zones. In this context, we examined the ontogeny of peptidergic cell populations expressing *Crh*, *Trh*, and *Ghrh* mRNAs in the mouse hypothalamus, comparing their distribution relative to the major progenitor domains characterized by molecular markers such as *Otp*, *Sim1*, *Dlx5*, *Arx*, *Gsh1*, and *Nkx2.1*. All three neuronal types originate mainly in the peduncular paraventricular domain and less importantly at the terminal paraventricular domain; both are characteristic alar *Otp/Sim1*-positive areas. *Trh* and *Ghrh* cells appeared specifically at the ventral subdomain of the cited areas after E10.5. Additional *Ghrh* cells emerged separately at the tuberal arcuate area, characterized by *Nkx2.1* expression. *Crh*-positive cells emerged instead in the central part of the peduncular paraventricular domain at E13.5 and remained there. In contrast, as development progresses (E13.5–E18.5) many alar *Ghrh* and *Trh* cells translocate into the alar subparaventricular area, and often also into underlying basal neighborhoods expressing *Nkx2.1* and/or *Dlx5*, such as the tuberal and retrotuberal areas, becoming partly or totally depleted at the original birth sites. Our data correlate a topologic map of molecularly defined hypothalamic progenitor areas with three types of specific neurons, each with restricted spatial origins and differential migratory behavior during prenatal hypothalamic development. The study may be useful for detailed causal analysis of the respective differential specification mechanisms. The postulated migrations also contribute to our understanding of adult hypothalamic complexity.

## Introduction

Studies on the ontogeny of peptidergic neurons in the rodent hypothalamus are relatively abundant, especially in the rat, although they provide variable and contradictory data (Daikoku et al. 1984; Grino et al. 1989; Burgunder and Taylor 1989; Rodier et al. 1990; Okamura et al. 1991; Baram and Lerner 1991; Burgunder 1991; Keegan et al. 1994). In general, these studies are based on the columnar-neurogenetic embryologic conception of Altman and Bayer (1986), which was based on the now outdated columnar model of Herrick (1910), inconsistent with gene patterns, and essentially offered conjectures about the areas where cell populations arise (see “Discussion”). In the meantime, the prosomeric model has been postulated as an alternative conceptual scenario for hypothalamic development (different length axis), held to be consistent with accrued gene expression evidence (Shimogori et al. 2010; Martinez et al. 2012; Puelles et al. 2012). This model has allowed very precise definition of diverse progenitor domains, each characterized by a differential molecular code. Following our earlier developmental analysis of somatostatin-expressing neurons within this model (Morales-Delgado et al. 2011), we address here the developmental topology of neurons producing corticotrophin-, thyrotrophin-, and growth hormone-releasing hormones (CRH, TRH, GHRH), mapped relative to the recently defined hypothalamic primary progenitor domains where they apparently originate (Shimogori et al. 2010; Puelles et al. 2012). These peptides are synthesized by neuroendocrine cells located mainly in the hypothalamus and the adjacent preoptic area. Their projections influence, respectively, the secretion of somatotroph, corticotroph, and thyrotroph cells of the adenohypophysis. However, these neuropeptides are also synthesized by non-hypophysiotropic neurons both inside and outside of the hypothalamus, which activate other functional systems (Hökfelt et al. 1975; Johansson et al. 1981; Bruhn et al. 1987; Tsuruo et al. 1987; Merchenthaler et al. 1988; Keegan et al. 1994; Lantos et al. 1995; Markakis and Swanson 1997; Petersenn and Schulte 2000; Armstrong 2004; Wang et al. 2011).

**Fig. 1:**
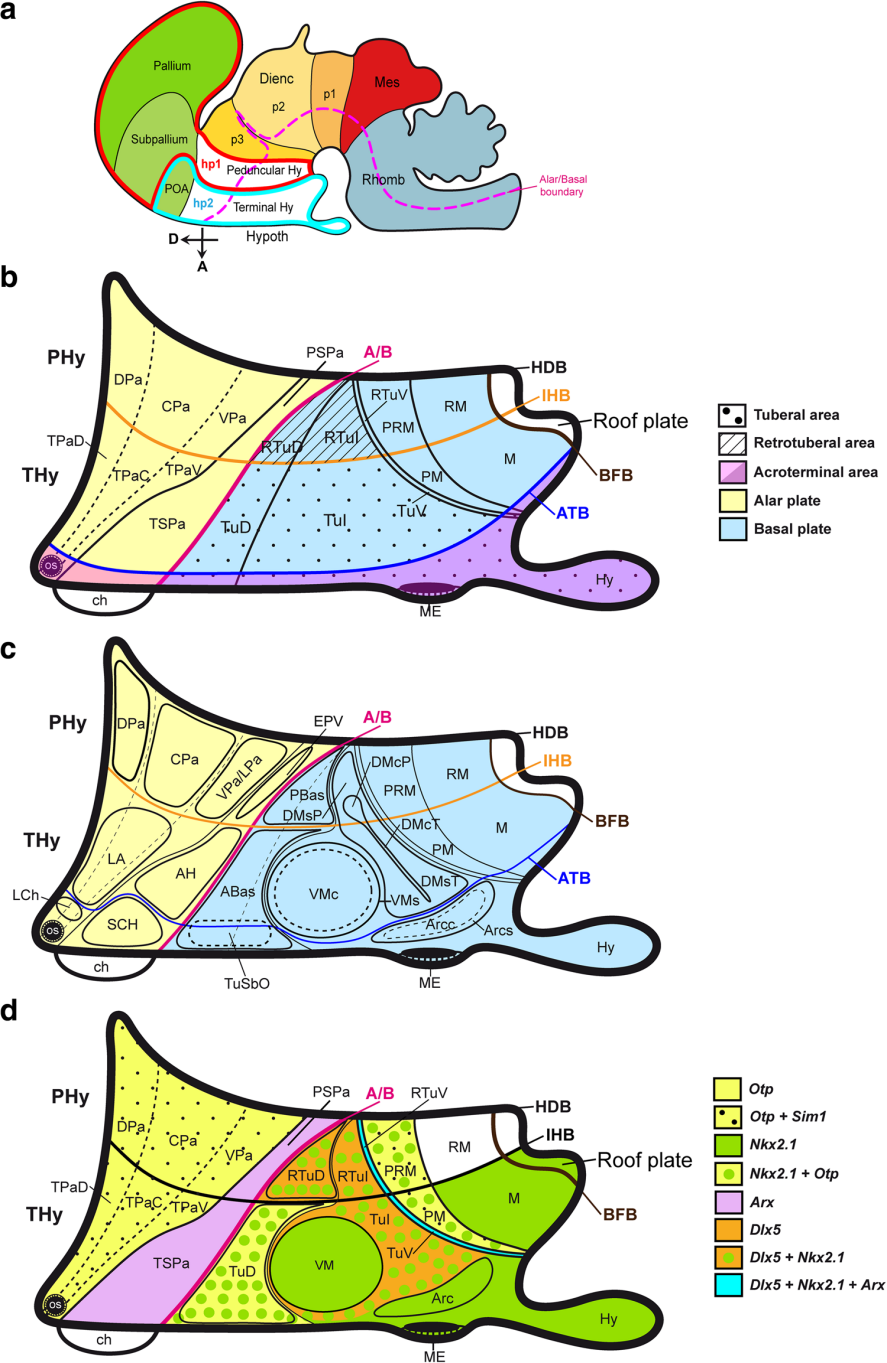
Schema of the forebrain representing the general position, morphologic organization and principal subdivisions of the hypothalamus, slightly modified from Morales-Delgado et al. (2011). The rostral (A) and dorsal (D) spatial directions are indicated. **a** Schema of the prosomeric model, showing the main neuromeric transverse subdivisions (hp2, hp1, p3, p2, p1). The hypothalamic area is highlighted in *white*, and is subdivided into peduncular and terminal moieties (PHy, THy), which, taken jointly with their respective dorsal telencephalic annexes, belong to the hypothalamic prosomeres 1 and 2, respectively (hp1 outlined in *blue*, and hp2 in *red*). **b** Schema of the fundamental hypothalamic progenitor areas distributed across the dorsoventral and anteroposterior dimensions. The longitudinal alar/basal boundary (A/B), and the intrahypothalamic (IHB) and acroterminal (ATB) borders are indicated, respectively, as *thick pink*, *orange*, and *blue lines*. Alar territories are seen on the *left* (*yellow*) and basal territories on the *right* (*blue*). The alar hypothalamus appears divided into the paraventricular (TPa/PPa) and subparaventricular (TSPa/PSPa) areas (each pair of areas refers to THy and PHy components of a longitudinal zone), plus corresponding acroterminal subregions. The paraventricular area shows a general tripartition into dorsal, central, and ventral parts (TPaD, TPaC, TPaV, DPa, CPa, VPa). The basal hypothalamus is also divided dorsoventrally into the large tuberal/retrotuberal (Tu/RTu) area, the perimamillary/periretromamillary (PM/PRM) area, and the mammillary/retromamillary (M/RM) area, plus the corresponding acroterminal subregions. The THy/PHy parts of the hypothalamic floor lie underneath (*white*). Moreover, the Tu/RTu region is subdivided into three dorsoventral parts: TuD/RTuD, TuI/RTuI, and TuV/RTuV. **c** Map of the main alar and basal hypothalamic nuclei represented upon the diagram in **b**. **d** Schematic *color*-coded map of the reference gene expression patterns used in our study, represented upon the same diagram. Details about overlaps occurring between given gene patterns are found in the legend. For abbreviations see the list

The recently updated prosomeric model holds that the hypothalamus is a rostral forebrain entity, ventral to the telencephalon, and rostral to the diencephalon proper, which is subdivided dorsoventrally into alar, basal, and floor longitudinal domains and separates rostrocaudally into two transverse regions called terminal hypothalamus (THy) and peduncular hypothalamus (PHy) (Fig. 1a). The lateral and medial forebrain bundles and the fornix tract (composing the cerebral peduncle) course dorsoventrally along PHy, which is also characterized by the generation of highly characteristic structures such as the main paraventricular nucleus, the retromamillary area and the migrated subthalamic nucleus (Puelles et al. 2012). On the other hand, the THy is traversed dorsoventrally by the hypophysotropic projections targeting the median eminence and neurohypophysis, and contains the massive tuberal and mamillary regions, as well as the evaginated optic vesicle and associated supraoptic, suprachiasmatic, and retrochiasmatic neuronal formations. The THy includes a singular rostromedian subdomain recently named acroterminal area (Puelles et al. 2012). This shows unique alar and basal specializations, such as the lamina terminalis (and related vascular organ), suprachiasmatic, and chiasmatic alar areas, and the anterobasal, arcuate, median eminence, and infundibular/neurohypophysial basal areas. Based on genoarchitectonic and fate map studies, the preoptic area is currently excluded from the hypothalamus, being ascribed instead to the telencephalic subpallium (Bulfone et al. 1993; Rubenstein et al. 1998; Puelles et al. 2000, 2004, 2012; Flames et al. 2007; Gelman et al. 2011; Bardet et al. 2010; Medina and Abellan 2012). The boundary of the hypothalamus with the telencephalon is presently defined molecularly by the ventral edge of the subpallial domain expressing *Dlx*, *Arx*, and *Mash1* genes.

Differential expression of several developmental genes abundantly demonstrates the existence of a distinct dorsoventral pattern across the hypothalamus, typically defining superposed longitudinal domains across both THy and PHy, which share given molecular markers (Shimogori et al. 2010; Puelles et al. 2012). For instance, the dorsal-most alar longitudinal domain is represented by the *hypothalamic paraventricular area* (Pa), an *Otp/Sim1*-positive and *Dlx/Arx*-negative domain, where most intrinsic neurons are glutamatergic (Puelles and Rubenstein 2003; Puelles et al. 2004, 2012; Shimogori et al. 2010; Morales-Delgado et al. 2011). Immediately underneath, there appears the subparaventricular area, a likewise alar domain expressing instead *Dlx, Arx, Isl1, Vax1*, and *Gad67*, where neurons are mainly gabaergic (loc. cit.; Pa; SPa; Fig. 1b).

The basal hypothalamus is largely distinguished from the alar counterpart by expression of *Nkx2.1* in the mantle layer, and *Shh* in the ventricular zone. It is particularly extensive dorsoventrally within the THy, where the classical *tuberal* and *mamillary* hypothalamic regions are found to be superposed one upon the other. The basal PHy shows analogous, though less massive, longitudinal domains, identified as *retrotuberal* and *retromamillary* regions, respectively (Tu; RTu, M; RM; Fig. 1b). The Tu territory further subdivides dorsoventrally into dorsal, intermediate and ventral progenitor subdomains (TuD, TuI, TuV), and similar RTuD, RTuI and RTuV subdomains can be identified within the neighboring RTu area (Fig. 1b). The underlying mamillary region is subdivided dorsoventrally into molecularly distinct perimamillary and mamillary areas (PM; M), which are, respectively, continuous caudalward with periretromamillary and retromamillary areas (PRM, RM; Fig. 1b; Puelles et al. 2012). The hypothalamic floor plate has been reformulated in the updated prosomeric model as being restricted to the retromamillary and mamillary neighborhoods, characterized by epichordal expression of marker genes such as *Shh, Ntn1, Lmxb1, Foxa1*, and *Nr4a2* (Puelles et al. 2012; Allen Brain Atlas).

The diversity of molecular profiles of the observed hypothalamic progenitor domains leads to the hypothesis that differentiation of specific types of neuropeptide-producing neurons is instructed positionally by a network of morphogen signals and transcription factors, which collectively specify the production of each phenotype. As occurs in other parts of the brain, regionally produced neuronal types subsequently intermix within the local mantle layer, and alternatively may migrate radially or tangentially, acquiring as differentiated cell types either aggregated or dispersed configurations at more or less distant adult sites relative to their origins (Morales-Delgado et al. 2011).

In the present report, we identified the specific neuroepithelial origins of CRH, TRH, and GHRH hypothalamic neurons, and followed them through intermediate developmental stages to apparently definitive locations, by using in situ hybridization analysis of both the progenitor landscape and the postmitotic populations under study. Our results indicate that the origins of a given population may be multiple, and tangential migrations often represent a salient aspect of their developmental pattern.

## Materials and methods

### Mouse embryos

All experimental protocols, handling use and care of mice were conducted in compliance with the current normative standards of the European Community (86/609/EEC), the Spanish Government (Royal Decree, 1201/2005; Law 32/2007), and the approval of University of Murcia Committee for Animal Experimental Ethics. For the present research, at least three Swiss albino mouse embryos per stage were collected at different embryonic days (E) after fertilization: 9.5, 10.5, 11.5, 12.5, 13.5, 15.5, and 18.5. The day of the vaginal post-coital plug formation was regarded as embryonic day 0.5 (E0.5). Embryos were separately staged according to the precise Theiler criteria (Theiler 1989). *Otp*−/– mice C57BL/6 embryos were produced from the heterozygous line kindly provided by Acampora and Simeone (Acampora et al. 1999).

### Tissue processing

Embryos from E9.5 to E13.5 were killed by decapitation and their heads were immersion-fixed in phosphate-buffered 4 % paraformaldehyde (0.1 M PB; pH 7.4) at 4 °C for 24 h. E15.5 and E18.5 embryos were anesthetized on ice and perfused transcardially with PB and the fixative solution. The brains were then dissected out and post-fixed for 24 h at 4 °C. Afterward, embryonic brains were transferred to 30 % sucrose in 0.1 M phosphate-buffered saline (PBS) solution for 24 h at 4 °C and then embedded in 15 % gelatin/20 % sucrose at 37 °C until they sank. The gelatin blocks were frozen for 2 min in isopentane at −55 °C and stored at −80 °C. Serial 14 μm-thick sections were cut in either the sagittal and transverse planes across the hypothalamus using a cryostat (Leica CM3500 S), collected as parallel series on SuperFrost Plus slides (Menzel-Gläser, Braunschweig, Germany), and reserved at −20 °C until use. *Otp*−/− mice embryo brains of various stages were embedded in 4 % agarose, cut 100 μm-thick with a vibratome in horizontal plane, and processed as floating sections for in situ hybridization of *Ghrh,* according to the protocol of Shimamura et al. (1995).

### Reverse transcription-polymerase chain reaction (RT-PCR)

*Arx, Crh, Ghrh, Gsh1, Sim1*, and *Trh* cDNA fragments were obtained by reverse transcription (RT). RNA was individually extracted with Trizol reagent (Invitrogen, Carlsbad, CA, USA) from fresh dissected brains of *Mus musculus* embryos at stages E10.5, 12.5, and 14.5. The RNA was treated with DNase I (Invitrogen) for 15 min at room temperature (RT), and then the enzyme was inactivated at 65 °C. RNA samples were then retro-transcribed into single-stranded cDNA with Superscript III reverse transcriptase and oligo dT anchored primers (Invitrogen, SuperScript First-Strand Synthesis System for RT-PCR). The resulting first-strand cDNA (0.5 μl of the reverse transcription reaction) was used as a template for PCR, performed with *Taq* polymerase (Promega, Madison, WI, USA) and specific primers for *Arx, Crh, Ghrh, Gsh1, Sim1*, and *Trh* mRNAs. The PCR conditions used were an initial denaturation step at 94 °C for 5 min, then 35 cycles [30 s at 94 °C, plus 1 min at Tm temperature (58 °C), and 1 min at 72 °C], followed by 10 min at 72 °C. The PCR products were cloned into pGEM-T Easy Vectors (Promega) and sequenced (SAI, University of Murcia). Primers: MArxF: 5′-CTGAGGCTCAAGGCTAAGGAG-3′, MArxR: 5′-GGTTTCCGAAGCCTCTACAGTT-3′; MCrhF: 5′-ACCTACCAAGGGAGGAGAAGAG-3′, MCrhR: 5′-GATGACTCCCATCTGCTTTTTC-3′; MGhrhF: 5′-GTCCCACCCAGGAGTGAAGG-3′, MGhrhR: 5′-AGCTGAAGCAGAAGTAACAG-3′; MGsh1F: 5′-AAGGCAAAGGCAGTAACCA-3′, MGsh1R: 5′-TGAAGGGGGTTTAGAGCGTA-3′; MSim1F: 5′-TATACTGCCTTTGGGGAGAGAA-3′, MSim1R: 5′-CTACCCGTACAACCTTTGTG-3′; MTrhF: 5′-CTGCCTTAGATTCCTGGATCAC-3′, MTrhR: 5′-GGAGGATGCGCTGAAGTTATAC-3′.

### In situ hybridization

Sense and antisense digoxigenin-UTP-labeled riboprobes for mouse *Arx, Crh, Dlx5*, *Ghrh*, *Gsh1*, *Nkx2.1,* *Otp*, *Sim1*, and *Trh* were synthesized following the manufacturer’s recommendations (Roche Diagnostics S.L., Applied Science, Barcelona, Spain) and applying specific polymerases (Fermentas, Madrid, Spain). Plasmid information is provided in Table 1. The hybridizations on cryosections were performed as described by Hidalgo-Sánchez et al. (2005). Hybridizations were carried out overnight at 72 °C (for *Otp* at 68 °C). RNA-labeled probes were detected by an alkaline phosphatase-coupled anti-digoxigenin antibody (diluted 1:3.500; Roche Diagnostics, Manheim, Germany), and the compound nitroblue tetrazolium/5-bromo-4-chloro-3-indolyl phosphate (NBT/BCIP; Roche Diagnostics) was used as a chromogenic substrate for the alkaline phosphatase reaction. Exposure times ranged between 1 and 2 days. No specific signal was obtained with sense probes in adjacent representative sections (data not shown).

**Table 1:** Used probes and their principal characteristics

Gene symbol	NCBI accession no.	Size (bp)	Positions	Linearization enzyme/polimerase	Publication/laboratory
*Arx*	NM_007492.3	920	1,691–2,611	*Nde*I/T7	Present results
*Crh*	NM_205769.1	920	144–1,063	*Nco*I/Sp6	Present results
*Dlx5*	NM_010056.2	1180	106–1,285	*Sph*I/Sp6	Morales-Delgado et al. (2011)
*Ghrh*	NM_010285.2	380	85–464	*Nde*I/T7	Present results
*Gsh1*	NM_008178.2	811	805–1,615	*Nco*I/Sp6	Present results
*Nkx2.1*	NM_009385.2	2216	597–2,813	*Sal*I/T3	Rubenstein
*Otp*	NM_011021.2	412	179–592	*Eco*R1/Sp6	Simeone
*Sim1*	NM_011376.3	726	5,827–6,552	*Nco*I/Sp6	Present results
*Trh*	NM_009426	960	309–1,268	*Nco*I/Sp6	Present results

### Imaging

The acquisition of entire-slide digital images was performed with a ScanScope CS digital slide scanner (Aperio Technologies, Vista, CA, USA). After scanning, the visualization and capture of images of adjacent labeled sections were carried out by using the software Aperio ImageScope. In all cases, contrast and focus of single consecutive photomicrographs were corrected, artificially pseudocolored (from dark blue to red or light blue) and superimposed by applying Adobe Photoshop CS3 software (Adobe Systems Inc., San José, CA, USA).

## Results

### Generalities of hypothalamic regionalization

In our analysis, we followed the nomenclature and genoarchitectonic subdivisions of the developing mouse hypothalamus postulated in the recently revised prosomeric model; the latter was briefly sketched in the “Introduction” and Fig. 1a–c; note that the paraventricular area can be subdivided dorsoventrally into dorsal, central, and ventral subareas (Fig. 1b; Puelles et al. 2012; see also the *Allen Developing Mouse Brain Atlas*, http://developingmouse.brain-map.org/). Insofar as the diverse alar and basal progenitor domains have each a characteristic molecular profile (see below), it is possible to characterize precisely the sites where given peptidergic cell types first differentiate. Similarly, any tangential cell displacements can be followed step by step the apparent movements, even if the conclusions remain tentative as long as experimental corroboration of the migration is pending.

#### Early developmental molecular demarcation of primary histogenetic areas of the hypothalamus

**Fig. 2:**
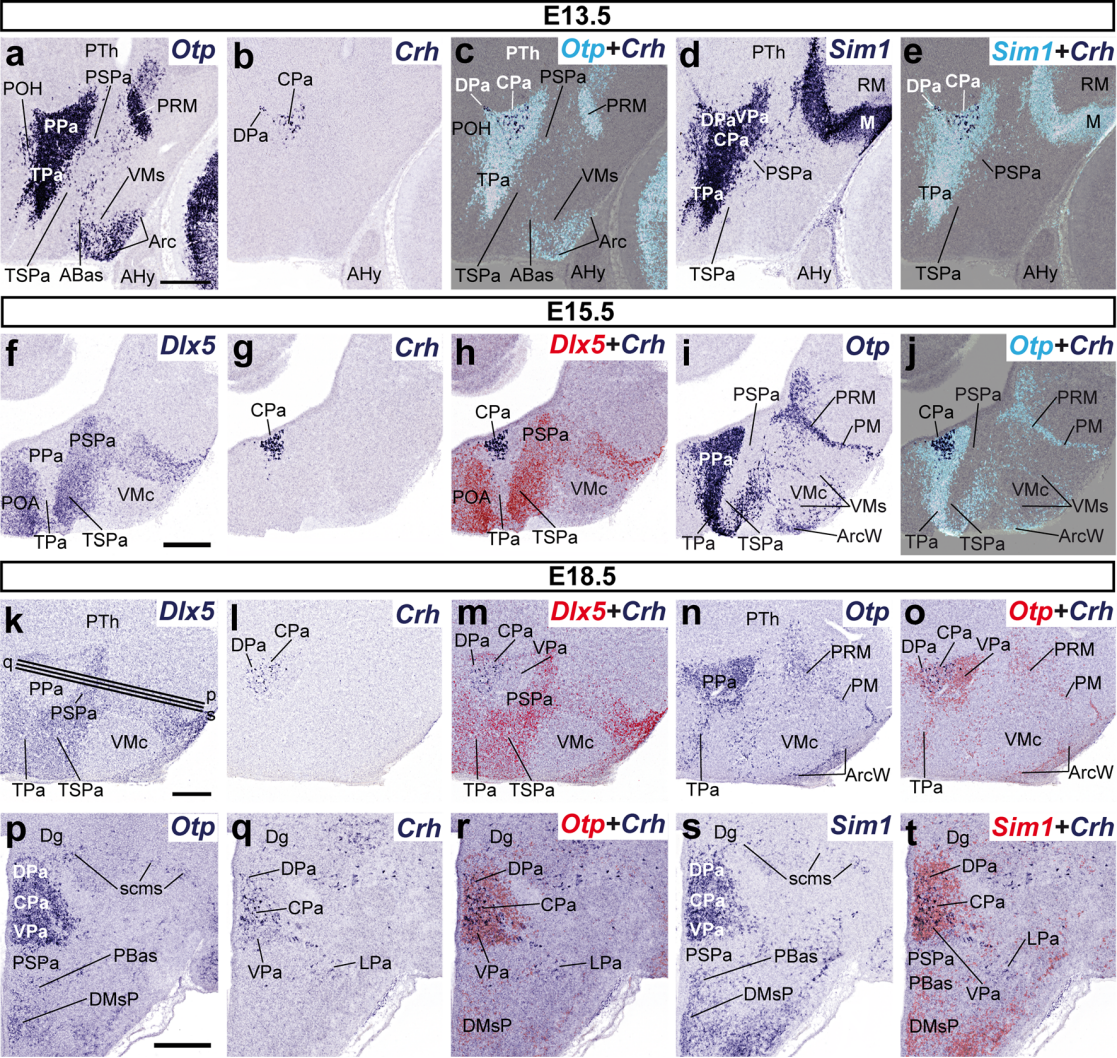
Parasagittal (**a**–**o**) and transverse (**p**–**t**) sections through the hypothalamus of E13.5 (**a**–**e**), E15.5 (**f**–**j**), and E18.5 (**k**–**t**) embryos at different levels, comparing in each case the presence of *Crh*-positive (*Crh*+) cells with expression of the indicated reference markers (*Otp*, *Sim1*, *Dlx5*). Corresponding digital overlaps with *pseudocolored* reference genes and *Crh* signal are marked with a *plus* symbol. Transverse section planes (**p**, **q**, **s**) are shown in **k**. *Scale* *bars* 300 μm

**Fig. 3:**
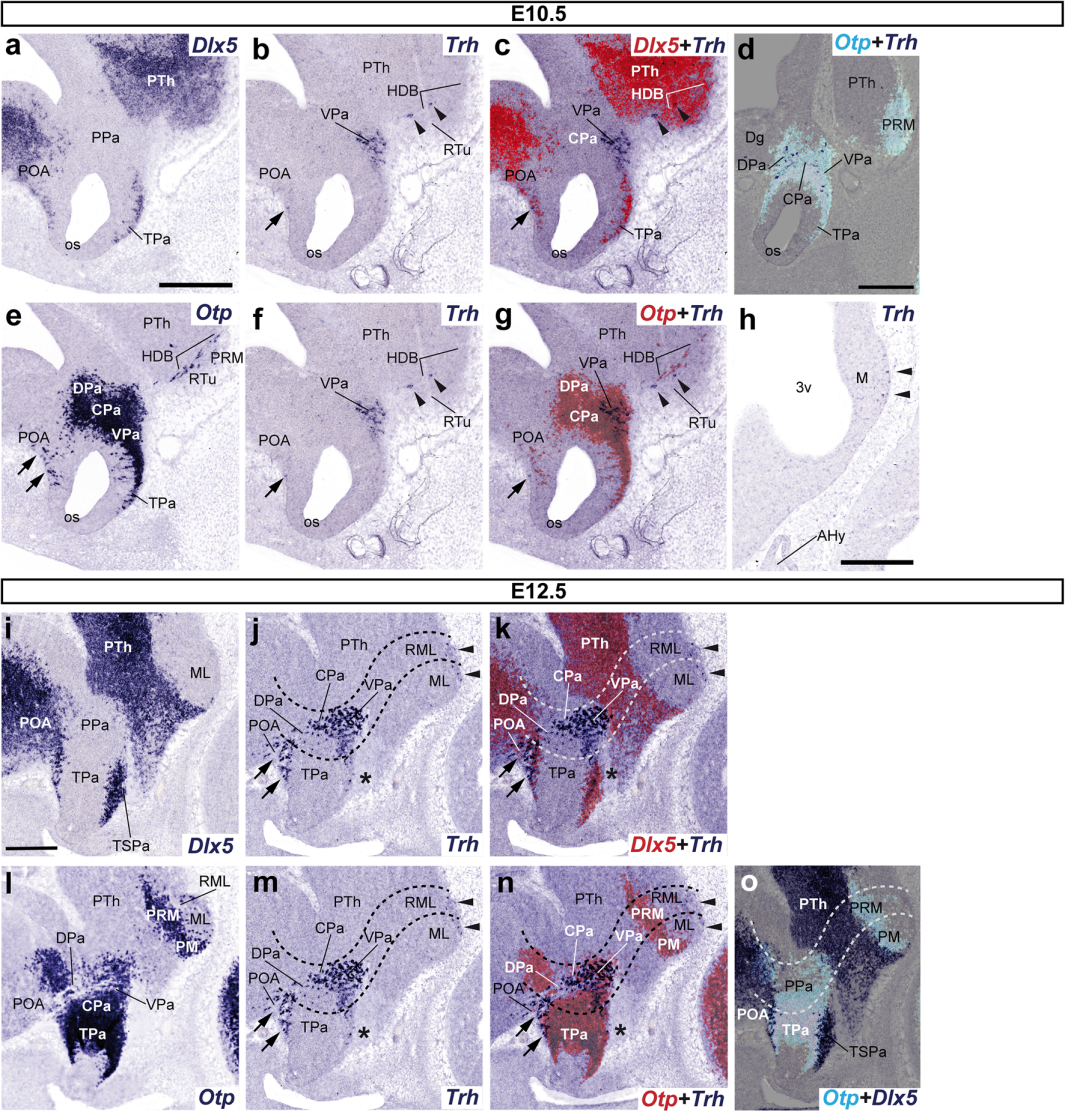
Sets of adjacent parasagittal sections from E10.5 (**a**–**h**) and E12.5 (**i**–**o**) embryos taken at lateral (**a**–**g**, **i**–**o**) and paramedian (**h**) levels correlate the presence of *Trh*+ cells with the indicated reference markers (*Otp*, *Dlx5*). Corresponding digital overlaps with *pseudocolored* reference genes and *Trh* signal are marked with a *plus* symbol. Transverse PHy (caudal) and THy (rostral) boundaries are indicated with *dashed* *lines* in **j**, **k**, **m**–**o**. Note that some *Trh*+ cells present at the surface of the mamillary/retromamillary region (*arrowheads* in **h**, **j**, **k**, **m**, **n**), as well as others intermixed with apparently migrated *Otp*+ cells within the extensive *Dlx5* + POA, dorsal to the preopto-hypothalamic boundary (*arrows* in **b**, **c**, **e**–**g**, **j**, **k**, **m**, **n**). Few dispersed *Trh*+ cells are found in close proximity to the imprecise *Otp/Dlx5* (TPa/TSPa) interface (*asterisks* in **j**, **k**, **m**, **n**) and close to the hypothalamo-diencephalic boundary (*arrowheads* in **b**, **c**, **f**, **g**). *Scale* *bars* 300 μm

**Fig. 4:**
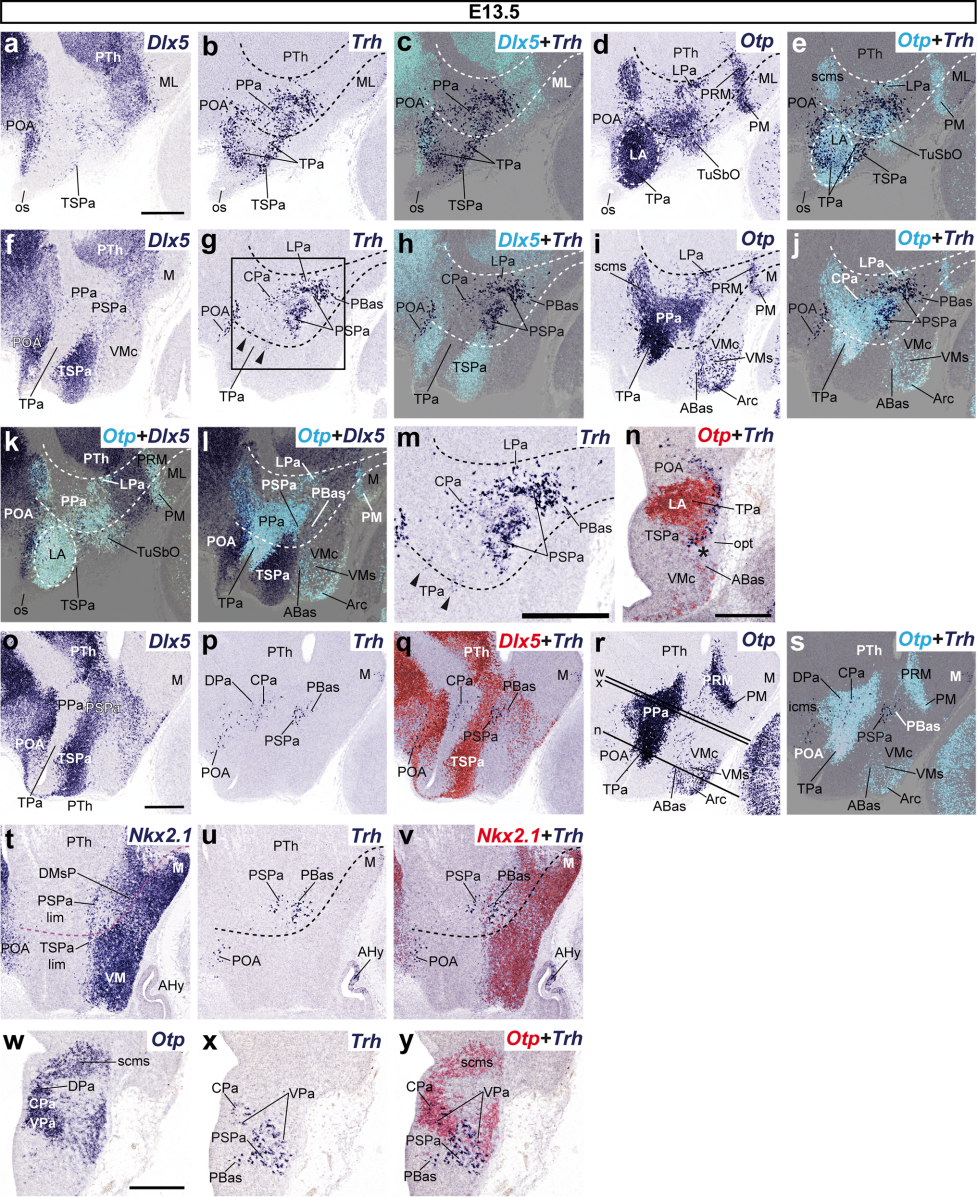
Parasagittal sections (**a**–**m**, **o**–**v**) through the hypothalamus of E13.5 embryos taken at lateral (**a**–**e**, **k**), intermediate (**f**–**j**, **l**), and median (**o**–**v**) levels, plus some transverse sections (**n**, **w**–**y**), correlating in each case the expression of indicated reference markers (*Dlx5, Nkx2.1, Otp*) with the presence of *Trh*+ cells. Corresponding digital overlaps with *pseudocolored* reference genes and *Trh* expression are marked with a *plus* symbol. **r** Rostrocaudal position of the transverse sections (**n**, **x**, **w**). **k**, **l** An overlapping expression of *Otp* and *Dlx5* genes. **m** Magnified image of the region outlined in **g**. The transverse hypothalamo-diencephalic and intrahypothalamic boundaries are represented with *dashed* *lines* (**b**–**e**, **g**–**j**, **k**–**m, t**–**v**). *Arrowheads* in **g** and **m** point to sparse *Trh*+ cells located next to the TPa/PPa borderline. Note abundant *Trh*+ cells in the *Dlx5* + TSPa, mixed with *Otp*+ cells (*asterisk* in **n**). *Scale* *bars* 300 μm

Consistently with described hypothalamic molecular regionalization (Shimogori et al. 2010; Puelles et al. 2012), we used specific gene markers to identify the dorsoventral hypothalamic subdivisions, which helped us to locate at early stages the progenitor domains associated to the first neuronal populations expressing *Crh*, *Ghrh*, and *Trh*. To this aim, we mapped the transcription factors *Otp* and *Sim1* at the Pa and PM/PRM areas, *Dlx5* at the SPa and Tu/RTu areas, *Arx* at the SPa, and *Nkx2.1* in the basal plate in general (Fig. 1d). Between E10.5 and E18.5, the separate Pa and PM/PRM domains are characterized by massive expression of *Otp/Sim1*; they showed practically no overlap with the telencephalic and hypothalamic *Dlx5/Arx*-positive domains (*Dlx5*+*/Arx*+; Figs. 1d, 2, 3o). The dorsal border of the *Otp*+*/Sim1*+ paraventricular area defines the hypothalamo-telencephalic boundary (Figs. 1d, 2a, d, 3o), held to coincide with a longitudinal plane that separates the telencephalic *stria terminalis* tract from the hypothalamic *stria medullaris* tract (Puelles et al. 2012). Throughout this period of development, *Nkx2.1* was widely expressed in the hypothalamic basal plate (with the exception of retromamillary region), as well as in the preoptic, diagonal, and pallidal parts of the telencephalic subpallium (Figs. 1d, 4t, 9y). The longitudinal alar/basal boundary (A/B; Fig. 1) is delineated by the abutting basal *Nkx2.1*- and alar *Arx/Dlx5*-expressing domains. Variations of these patterns will be detailed below.

### Ontogeny of *Crh*-positive cells

The *Crh* expression pattern is quite simple and does not vary significantly through the studied period. At E13.5, we found a localized caudal subpopulation of periventricular *Crh*+ cells in the central part of the peduncular Pa domain (prospective CPa nucleus; Figs. 1c, 2b); only very few such cells appeared in the dorsal part of this complex, and none in the ventral part (prospective DPa and VPa nuclei). No *Crh* expression was detected at earlier stages (data not shown). The peduncular Pa, the largest part of the Pa area, is an *Otp*+*/Sim1*+ trapezoidal domain flanked ventrally and dorsally by *Dlx5*-expressing domains (Figs. 1d, 2a, d, f, i). It is well known that the adult Pa complex contains various small- and large-celled neuronal aggregates. At E15.5, the distribution of *Crh* cells practically unchanged, being restricted to an abundant, dense subpopulation of the alar CPa (Fig. 2f–j). Finally, sagittal and transverse sections through the E18.5 hypothalamus revealed that the population of *Crh*+ cells appears now more dispersed than before. *Crh*+ cells were detected mainly in the dorsal and central portions of peduncular Pa (DPa, CPa; Fig. 2k–t). A small number of *Crh*+ cells were located laterally in the intermediate stratum of the VPa (Fig. 2p–t). The terminal Pa area appeared always devoid of *Crh* cells.

### Ontogeny of *Trh*-positive cells

#### Majority of first Trh-expressing cells are located in the ventral subdivision of the peduncular paraventricular complex at E10.5 and E11.5

No *Trh* expression was detected at E9.5 (data not shown). *Trh*-expressing cells first appeared in the ventral part of the peduncular paraventricular complex (VPa) at E10.5 (Fig. 3a–g). Some superficial *Trh*+ cells were also located in the dorsal part of Pa, close to the boundary between the hypothalamus and the subpallial diagonal area (DPa; Dg; Fig. 3d). The neighboring *Dlx5*+ preoptic region also showed some *Trh*+ cells mixed with dispersed apparently migrated *Otp*+ cells (arrows; Fig. 3a–c, e–g). Separately, we also detected some *Trh*+ cells in the periretromamillary area (mixed with *Otp*+ neurons) (arrowheads; Fig. 3b, c, f, g) and superficially at the mamillary area (arrowheads; Fig. 3h). We observed no changes in this pattern at E11.5.

#### Trh-positive cells are dispersed ventrodorsally within the peduncular domain of the alar paraventricular complex at E12.5

The *Trh* expression pattern at E12.5 was generally consistent with the earlier pattern, with the addition of some new expression in the terminal paraventricular domain. However, the major group of *Trh*+ cells still appears in the VPa. This population also spreads to the suprajacent central subarea of Pa (CPa; Fig. 3j, k, m, n). In addition, *Trh*-expressing cells continued to be observed in the DPa, in close proximity to a larger group of such cells found within the *Dlx5* + POA, partially intermixed with preoptic *Otp*+ cells (arrows; Fig. 3i–n). The ventral subdomain of terminal Pa (TPaV) first showed at this stage a few *Trh*+ cells (asterisks; Fig. 3j, k, m, n). As at earlier stages, *Trh* cells also appeared sparsely at the surface of the mamillary/retromamillary complex (ML/RML; arrowheads; Fig. 3j, k, m, n).

#### Increase of alar Trh neuronal populations and appearance of posterobasal Trh cells at E13.5

At E13.5, more abundant *Trh*+ cells were found in the alar plate (PPa, TPa). In contrast to the previous stage studied, *Trh* signal is now widespread at the TPa. At this age, some *Trh* cells were also found within the PSPa, suggesting a ventralward migration (see below).

At the TPa domain, lateral sagittal sections highlight populations of superficial cells expressing *Trh* and *Otp,* mixed together within the characteristic ovoid primordium of the lateral anterior hypothalamic nucleus (LA); other laterally placed *Trh*+ cells were identified more dorsally within the extensive *Dlx5* + POA, located just above the preopto-hypothalamic boundary (LA, POA; Fig. 4a–e, k, n). At intermediate levels, we only detected few scattered *Trh*+ cells at the TPa/PPa limit (arrowheads; Fig. 4g, m). At the PPa domain, *Trh* cells were particularly abundant at the VPa medial subdomain, and extended in more lateral sections to the presumptive lateral paraventricular nucleus (LPa), a radially migrated derivative of the VPa found in front of the hypothalamo-prethalamic boundary (Fig. 4a–j, l, m). In addition, there was some *Trh* expression in the CPa, but scarcely any at the DPa. This distribution pattern was also evident in transverse sections (Fig. 4w–y). At periventricular levels of PPa, scarce *Trh*+ cells were mainly restricted to the central paraventricular subdivision (CPa), although occasional *Trh*+ cells were also distributed in its dorsal and ventral parts (DPa, VPa; Fig. 4o–y).

Sagittal and transverse sections showed additional *Trh*+ cells within the *Dlx5* + TSPa, mixed with *Otp*+ cells (Fig. 4a–e; asterisk in Fig. 4n). A sizeable population of *Trh* cells was also found within the neighboring PSPa area in lateral sagittal sections at this stage; we believe that these SPa cells migrate ventralward from the VPa into the underlying basal plate, and are here observed transiently as they traverse the PSPa; at E13.5 some of them have already reached the dorsal area of the retrotuberal basal plate, settling within the PBas nucleus, which displays a mixed population of *Trh*+ and *Dlx5*+ cells (Fig. 4f–j, m, o–q, w–y). The *Trh*+ cells invading PBas typically penetrate a dorsorostral PBas area where *Nkx2.1* is practically not expressed (Fig. 4t–v). In more medial sagittal sections, two distinct *Trh*+ cell aggregates could be distinguished within the narrow PSPa: a rostral group was found adjacent to the intrahypothalamic boundary (IHB), whereas a separate caudal group appeared next to the hypothalamo-diencephalic boundary (HDB) (Fig. 4f–j, m).

#### Number of Trh cells in the hypothalamic basal plate increases at E15.5

At E15.5, we found in general that the basal populations of *Trh*+ cells in both the PHy and THy increase remarkably, in detriment of alar ones (Pa and SPa).

**Fig. 5:**
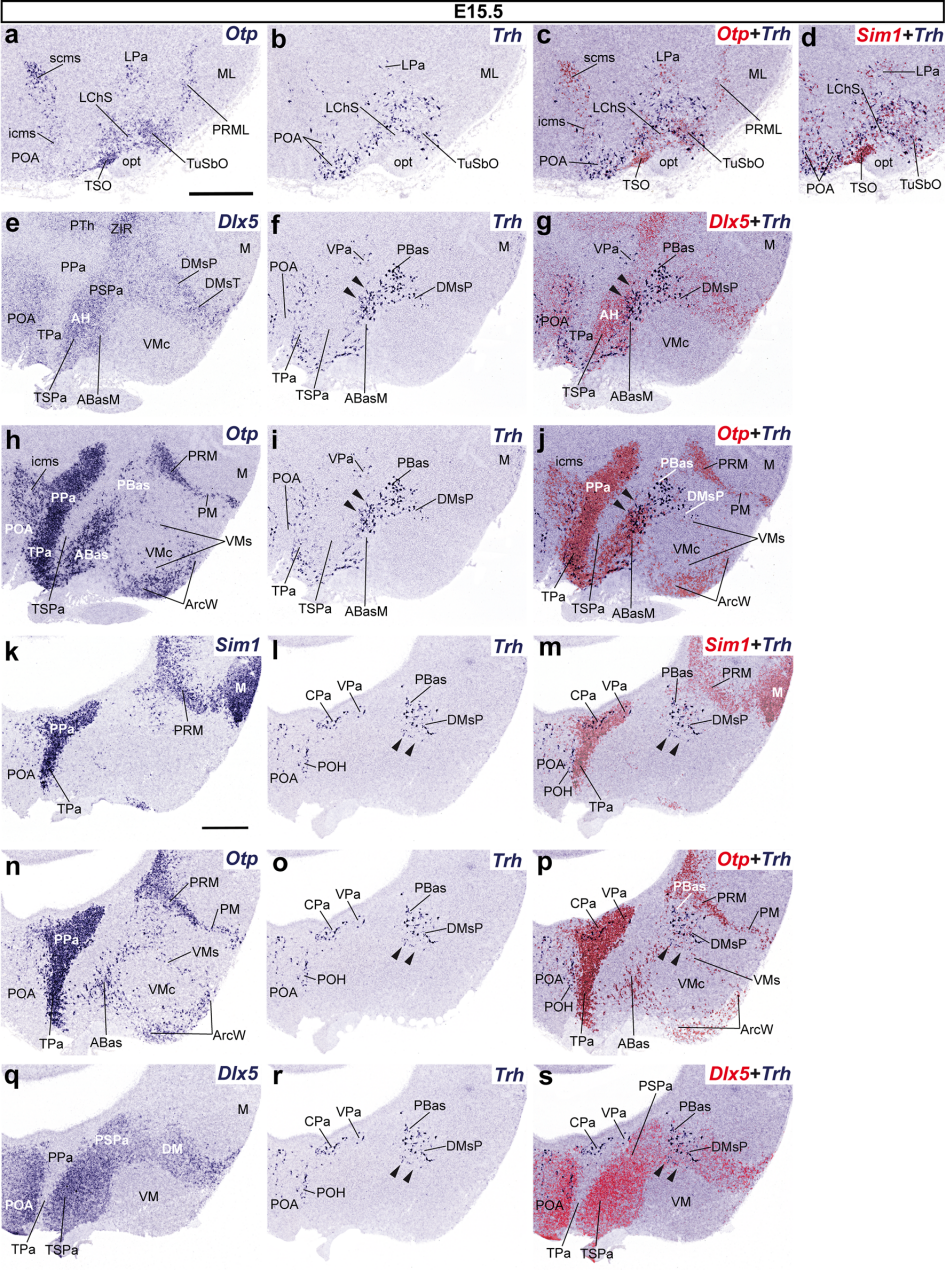
Parasagittal sections from an E15.5 embryo taken at lateral (**a**–**d**) and medial (**e**–**s**) levels, comparing in each case the expression pattern of the indicated reference genes (*Otp, Sim1, Dlx5*) with *Trh*+ cells. Corresponding digital overlaps with *pseudocolored* reference markers and *Trh* signal are marked with a *plus* symbol. Note that dispersed *Trh*+ cells lie within the borders of the *Otp*+*/Dlx5* + PBas territory (*arrowheads* in **f**, **g**, **i**, **j**), as well as within the shell of the ventromedial nucleus (VMs; *arrowheads* in **l**, **m**, **o**, **p**, **r**, **s**). *Scale* *bars* 300 μm

At the acroterminal TPa region, fewer *Trh*+ cells were present at the LA nucleus, though some *Trh*+ cells appeared intermingled with *Otp*+ and *Sim1*+ cells within the immature superficial laterochiasmatic nucleus (LChS; Fig. 5a–d). The latter was recently identified by Morales-Delgado (2012), and corresponds to a superficial subarea of the alar formation previously named *laterochiasmatic nucleus* (Puelles et al. 2012; Morales-Delgado 2012). This is an ovoid *Otp*+ neuronal aggregate lying immediately dorsal to the characteristic *Dlx5*+ suprachiasmatic nucleus (TSPa). From here, numerous *Trh*+ cells invade the rostral part of TSPa, without entering the primordium of the anterior hypothalamic nucleus (AH; Fig. 5e–j). In median sagittal sections, we observed an extensive population of dispersed *Trh*+ cells, located rostrally, close to the optic chiasm, which roughly corresponds to the tuberal ABas area (Fig. 5e–j) and few *Trh*+ cells in the shell of the ventromedial hypothalamic nucleus (VMs; arrowheads, Fig. 5k–s). *Trh*+ cells extend likewise into the basal *Sim1*+*/Otp*+ tuberal suboptic nucleus (TuSbO; Fig. 5a–d). We had not detected any suboptic *Trh*+ cells at E13.5.

Conversely, in the PHy we found relatively fewer *Trh*+ cells in the trapezoidal PPa domain, scattered within the LPa, VPa, and CPa (Fig. 5a–s), or in the PSPa (arrowheads; Fig. 5e–j), whereas a parallel increase was noted in the number of *Trh*+ cells in the peduncular basal hypothalamus (Fig. 5e–s). A dense group of *Trh*+ cells was still found at the PBas subdomain, also characterized at E15.5 by dispersed *Otp*+ or *Dlx5*+ cells (Fig. 5e–s). Ventrally to the PBas, we first observed *Trh*+ cells within the shell of the otherwise *Dlx5*+ peduncular dorsomedial nucleus (DMsP); in contrast to other basal places, here few of the *Trh*+ cells coincided topographically with the local *Otp*+/*Sim1*+ cells; this suggests that the latter probably translocated dorsalward from the adjacent PRM domain (Fig. 5e–p).

#### Trh expression pattern at E18.5

**Fig. 6:**
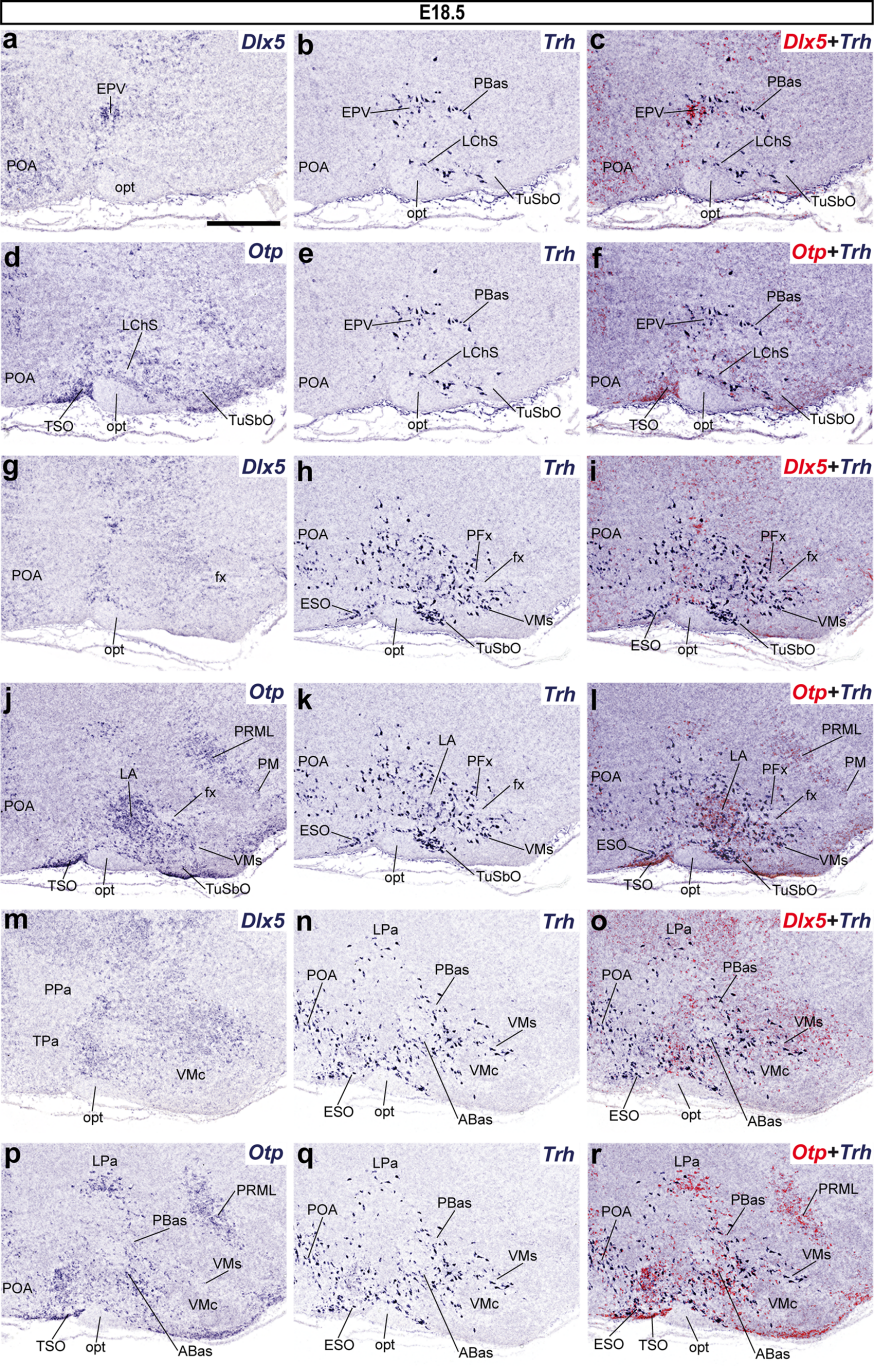
Series of parasagittal sections through the hypothalamus of an E18.5 embryo at lateral (**a**–**l**) and intermediate (**m**–**r**) levels, correlating the expression pattern of the indicated reference markers (*Otp, Dlx5*) with the presence of *Trh*+ cells. Corresponding digital overlaps with *pseudocolored* reference markers and *Trh* signals are marked with a *plus* symbol. *Scale* *bars* 300 μm

**Fig. 7:**
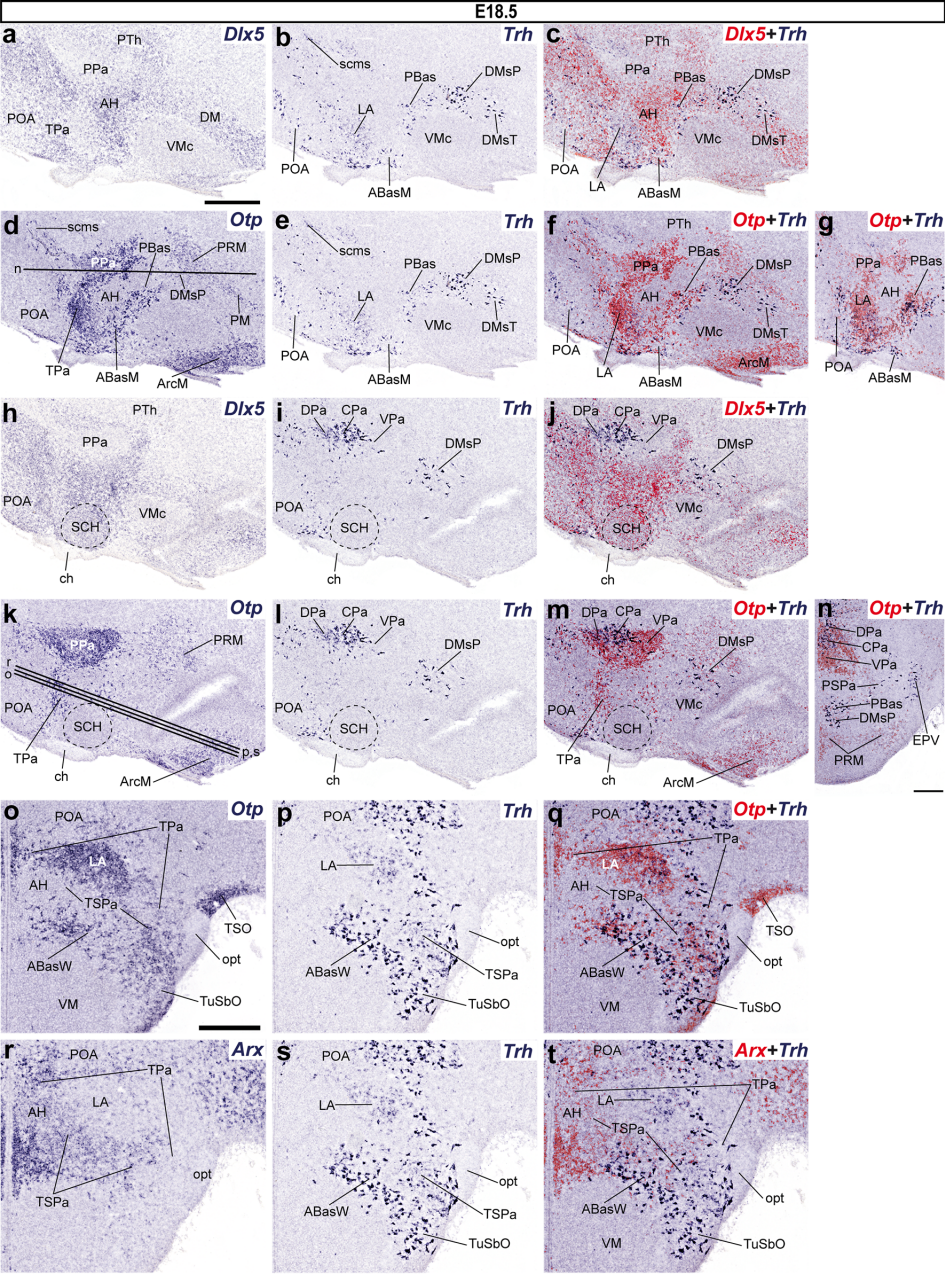
Medial parasagittal sections (**a**–**k**) and transverse sections at two different levels (**n**–**t**) through stage E18.5 mouse embryos, correlating in each case the expression pattern of the indicated reference markers (*Arx*, *Dlx5, Otp*) with the presence of *Trh*+ cells. Corresponding digital overlaps with *pseudocolored* reference genes and *Trh* expression are marked with a *plus* symbol. The transverse section planes shown (**n**, **o**, **p**, **r**, **s**) are identified at the **d** and **k** panels. *Scale* *bars* 300 μm

Overall, results at stage E18.5 largely repeated the *Trh* distribution observed at E15.5 although with increasing complexity and some additional *Trh* cell populations not found at E15.5. We will concentrate on these latter aspects. At the superficial stratum of the PSPa, we first identified a small group of peduncular *Trh*+ cells that partly correlate in position with the *Dlx5*+/*Otp*- *ventral entopeduncular nucleus* (EPV; Figs. 6a–f, 7n). This nucleus lies interstitially within the peduncle, approximately at the level where the optic tract arches superficially over it (Puelles et al. 2012). Moreover, fewer *Trh*-expressing cells were distinguished at the acroterminal LChS than previously (Fig. 6a–f). At intermediate levels of the PHy, *Trh*-expressing cells persist in the LPa primordium (Fig. 6m–r), whereas in THy similar cells appear in the lateral anterior hypothalamic nucleus (LA; Fig. 7a–g, o–t). Finally, at periventricular levels, the alar expression of *Trh* is restricted to the PPa. We identified a large population of *Trh*+ cells distributed within its dorsal and central portions (DPa, CPa), whereas its ventral portion (VPa), in contrast to earlier stages, is practically devoid of them (Fig. 7h–n).

On the other hand, in the basal plate, *Trh*+ cells were more numerous than previously in the *Otp*+ and *Dlx5*-*/Arx*-TuSbO territory. These suboptic cells lie very close to the VMs, in which some *Trh*+ cells were also present (Figs. 6g–l, 7o–t). We also detected a new alar aggregate of *Trh*+ cells, comprised by a small number of aligned *Trh*+ cells identified just dorsal to the characteristic *Otp*+ supraoptic nucleus (TSO); we identified it as the *episupraoptic nucleus* (ESO; Fig. 6d–r; Watson and Paxinos 2010). Another new *Trh* population is deeper and has a perifornical distribution (rostral part of PHy) at levels through the PSPa and the underlying PBas; it is intermixed with scattered *Otp*+ cells (PFx; Fig. 6g–l). *Trh* expression was also detected in several basal subdomains such as the ABas (wings included; ABasW), the PBas and the VMs (Figs. 6m–r, 7a–g, o–t). The shell portion of the *Dlx5*+ terminal dorsomedial nucleus (DMsT) first shows *Trh*+ cells at this stage; they are intermixed with *Otp*+ cells showing a different distribution, suggesting the latter may have migrated instead from the underlying perimamillary domain **(**DMsT; PM; Fig. 7a–f). The peduncular dorsomedial nucleus counterpart also contains *Trh*-expressing cells (DMsP; Fig. 7a–m).

### Ontogeny of *Ghrh*-positive cells

#### First Ghrh-expressing cells are restricted to the ventral subdivision of the peduncular paraventricular complex at E10.5

**Fig. 8:**
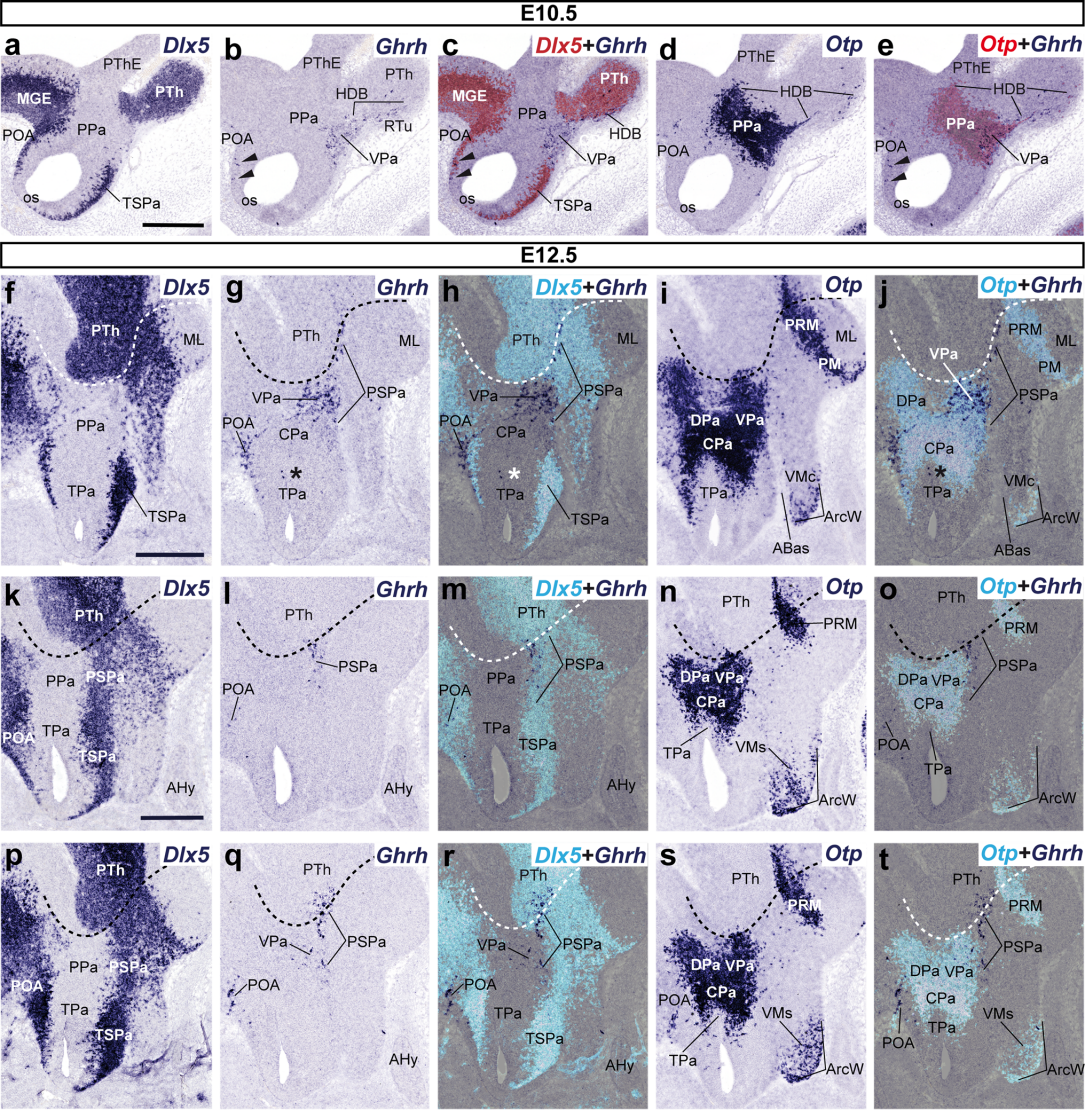
Lateral parasagittal sections from E10.5 (**a**–**e**) and E12.5 (**f**–**t**) embryos, correlating the presence of *Ghrh*+ cells with the indicated reference markers (*Otp*, *Dlx5*). Corresponding digital overlaps with *pseudocolored* reference markers and *Ghrh* cells are marked with a *plus* symbol. The transverse hypothalamo-diencephalic boundary is indicated with *dashed* *lines* in **f**–**t**. *Arrowheads* in **b**, **c**, **e** mark the presence of some dispersed *Ghrh*+ cells within the *Dlx5*+preoptic area, lying immediately dorsal to the preopto-hypothalamic limit and coinciding also with *Otp*-positive cells. *Asterisks* in **g**, **h**, **j** indicate some *Ghrh*+ cells in TPa. *Scale* *bars* 300 μm

There was no *Ghrh* mRNA expression at E9.5 (not shown). *Ghrh*+ neurons first appeared in the mantle of VPa at E10.5 (Fig. 8a–e). Some of these cells spread along the hypothalamo-prethalamic boundary (HDB; Fig. 8a–e). As found with the *Trh* expression pattern, the *Dlx5*+ preoptic region (overlying telencephalic subpallium) also contained at this stage some *Ghrh*+ cells mixed among apparently migrated *Otp*+ cells (arrowheads; Fig. 8b, c, e). The expression pattern of *Ghrh* showed no change at E11.5 (data not shown).

#### Ghrh expression is restricted to the alar PHy at E12.5

In general, the hypothalamic distribution of *Ghrh* mRNA remains mainly constrained to the ventral subdomain of the PPa (VPa) at E12.5. However, we also detected some *Ghrh*+ cells at the subjacent *Dlx5* + PSPa alar domain, which we interpreted as cells migrated from the overlying VPa. The labeled PSPa population is caudally contiguous with separate *Ghrh* expression in the prethalamus, appeared primarily at the zona incerta, beyond the HDB (PTh; Fig. 8f–t). A few *Ghrh*+ cells were also detected in the TPa (asterisks; Fig. 8g, h, j).

#### First appearance of Ghrh-positive cells in basal subdomains of the terminal and PHy at E13.5

**Fig. 9:**
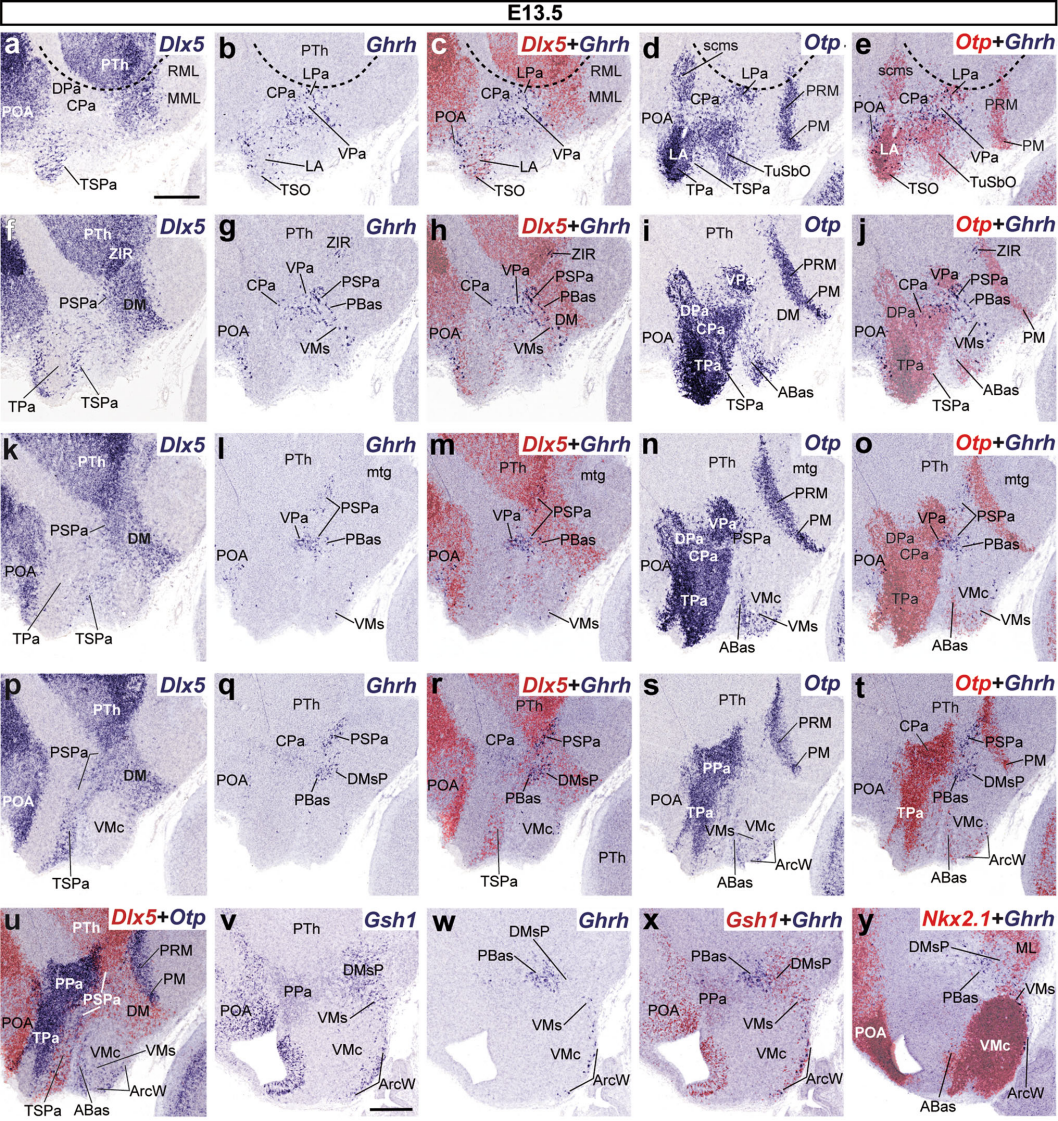
Pairs of adjacent parasagittal sections of E13.5 embryos taken at lateral (**a**–**j**), intermediate (**k**–**u**), and medial (**v**–**y**) levels, comparing in each case the expression pattern of the indicated reference genes (*Dlx5, Gsh1, Otp, Nkx2.1*) with the presence of *Ghrh*+ cells. Corresponding digital overlaps with *pseudocolored* reference markers and *Ghrh* signals are marked with a *plus* symbol. *Dashed* *lines* in **a**–**e** represent the hypothalamo-diencephalic boundary. *Scale* *bars* 300 μm

The main population is still concentrated in the peduncular VPa/PSPa areas at E13.5, but also extends now rostralward into the terminal paraventricular domain. Basal *Ghrh* cells first appear in both the THy and PHy. The *Ghrh* pattern at superficial levels of the mantle was on the whole comparable to that of *Trh* at this stage, although with relatively less *Ghrh*+ cells (compare Figs. 4, 9).

The *Ghrh*+ population was still abundant in the ventral PPa subdomain (VPa), with some components now detected more superficially in the mantle zone, within the incipient lateral paraventricular nucleus (LPa), also a derivative of VPa; a few dispersed *Ghrh*+ cells were also observed more dorsally in the CPa (Fig. 9a–j). The subjacent PSPa area shows now a larger number of *Ghrh* cells, particularly at deep mantle levels and close to the IHB and HDB limits (Fig. 9f–t). Dispersed *Ghrh*+ cells appeared now also in the correlative alar terminal areas (TPa/TSPa), particularly within the voluminous *Otp*+ lateral anterior nucleus and the terminal supraoptic nucleus (LA, TSO; Fig. 9a–e). These are interpreted as elements originated separately from the peduncular counterparts. Other *Ghrh*+ neurons were found mixed with *Otp*+ cells in the suprajacent *Dlx5* + POA, lying beyond the longitudinal subpallio-hypothalamic boundary (Fig. 9a–e). This may be a separate preoptic (telencephalic) origin. We also found non-hypothalamic *Ghrh*+ cells in the primordium of the *prethalamic* *zona* *incerta*, rostral part (ZIR), which is the ventralmost alar subregion of the prethalamus (Fig. 9f–j). This territory is rostrally continuous with the hypothalamic subparaventricular band (SPa) and shares a number of gene markers (e.g., *Dlx*, *Arx*).

The impression that *Ghrh*+ cells move at this stage out of PPa/TPa, passing ventralward across PSPa/TSPa, is reinforced by gradual appearance of *Ghrh*+ cells within neighboring basal hypothalamic areas (Fig. 9f–y). We found a number of *Ghrh*+ cells within the dorsal and intermediate subregions of the retrotuberal basal PHy (PBas, DMsP domains, respectively). The PBas is the dorsalmost longitudinal basal subdomain within PHy, characterized by the expression of *Dlx5* and *Gsh1* genes, but where *Nkx2.1* is less strongly expressed than elsewhere in the basal plate (Fig. 9p–y). In contrast, at the THy we found some scattered *Ghrh*+ cells at the subpial *wings* of the Arc and at the ventral part of the VM shell (ArcW; VMs; Fig. 9f–y). These are interpreted here as a separately originated arcuate basal population, which partly disperses laterally and under the VM (see below and “Discussion”). All these basal hypothalamic tuberal areas also contain cell populations expressing the *Otp*, *Gsh1*, and *Nkx2.1* markers (Fig. 9s–y).

#### Alar sources of *Ghrh*-expressing cells become depleted in favor of basal sites at E15.5 and E18.5, whereas new populations appear in the superficial hypothalamus

**Fig. 10:**
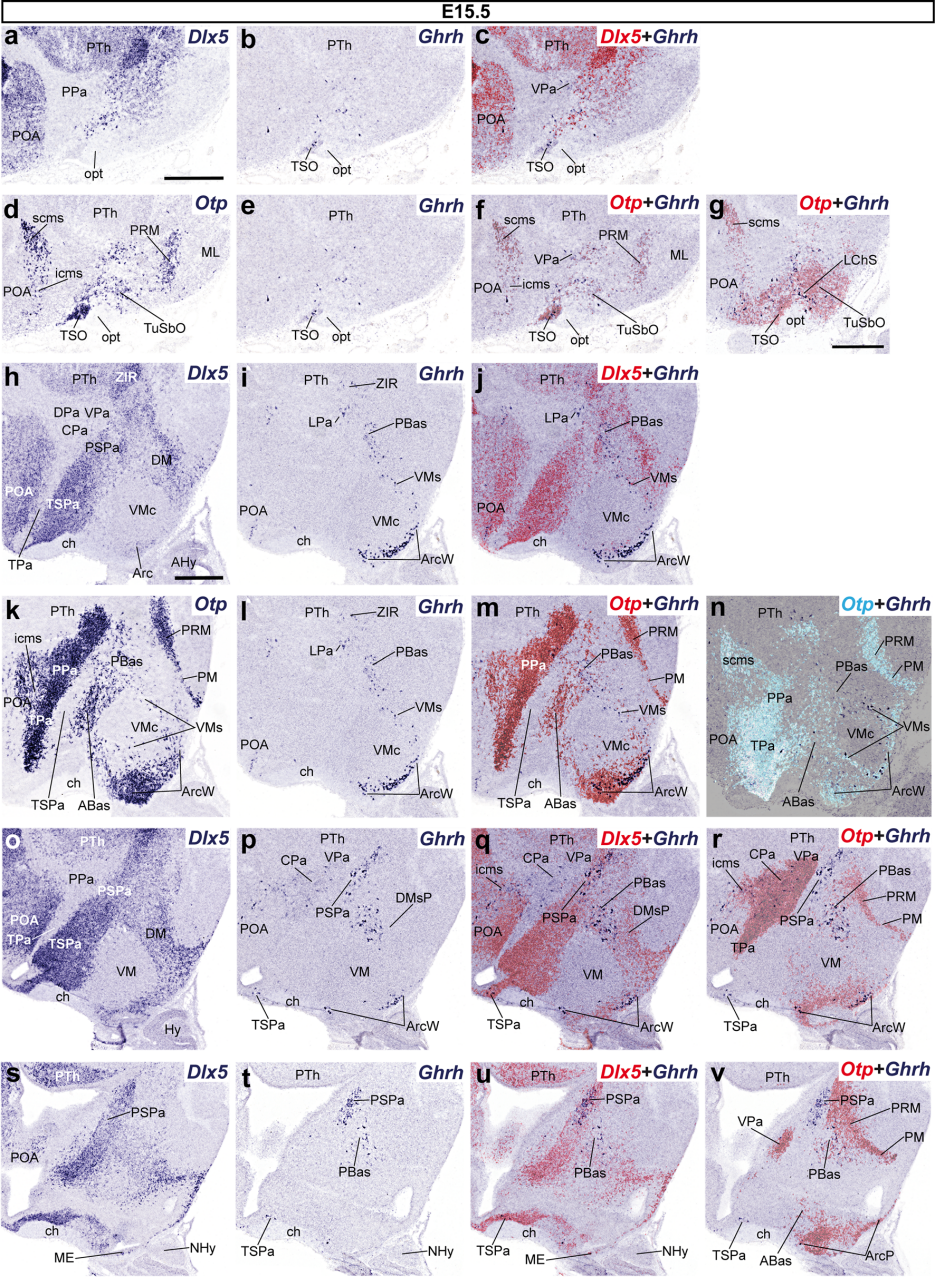
Series of parasagittal sections through the hypothalamus of E15.5 embryos taken at lateral (**a**–**g**), intermediate (**h**–**n**), and median/paramedian (**o**–**v**) levels, correlating the expression pattern of the indicated reference markers (*Otp, Dlx5*) with the presence of *Ghrh*+ cells. Corresponding digital overlaps with *pseudocolored* reference markers and *Ghrh* signals are marked with a *plus* symbol. *Scale* *bars* 300 μm

At E15.5, the distribution of *Ghrh* mRNA appeared comparable to that described at E13.5, except in the superficial hypothalamus. *Ghrh*+ cells were virtually absent in the alar VPa from E15.5 onwards; we only detected some dispersed *Ghrh* cells in its ventral and central portions (VPa, CPa; Fig. 10o–r). An additional small group characterizes the lateralized LPa (Fig. 10h–m). The underlying narrow PSPa still shows a relevant longitudinal contingent of *Ghrh*+ cells extending caudally back to the hypothalamo-prethalamic border; however, the aggregated cells found earlier next to the IHB within PSPa have disappeared (presumably moved into the basal region) (Fig. 10o–v). No *Ghrh*+ cells were observed at the deep periventricular levels of the TPa/TSPa areas (Fig. 10h–v), whereas some of them were found within the subpial terminal supraoptic nucleus (TSO), which is identified by a dense *Otp* signal and the absence of *Dlx5* expression (Fig. 10a–f). Ventrally to the optic tract, we also found some dispersed subpial *Ghrh*+ cells within the superficial laterochiasmatic (LChS) and tuberal suboptic (TuSbO) nuclei (Fig. 10g); the latter lies in the terminal basal region, though its cells presumably have migrated from the TPa domain. Other cells expressing *Ghrh* within the basal plate were present as before at peduncular (PBas and DMsP) and terminal (VMs and ArcW) areal subdomains. Such basal elements increased particularly within the ArcW (Fig. 10h–v).

**Fig. 11:**
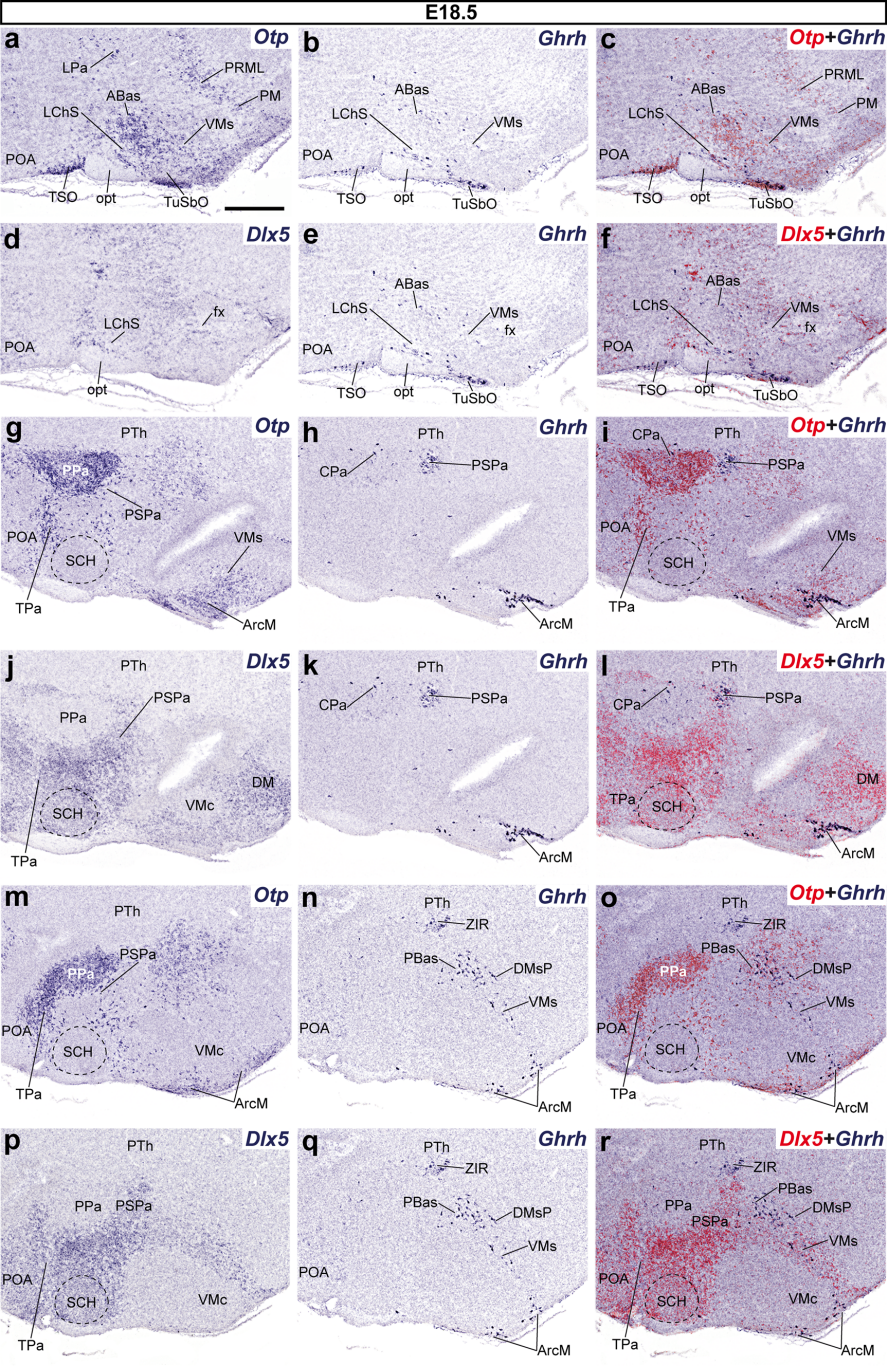
Series of parasagittal sections through an E18.5 embryo from lateral (**a**–**f**) to medial (**g**–**r**) levels, correlating the expression pattern of the indicated reference markers (*Otp, Dlx5*) with the presence of *Ghrh*+ cells. Corresponding digital overlaps with *pseudocolored* reference markers and *Ghrh* signals are marked with a *plus* symbol. *Scale* *bar* 300 μm

**Fig. 12:**
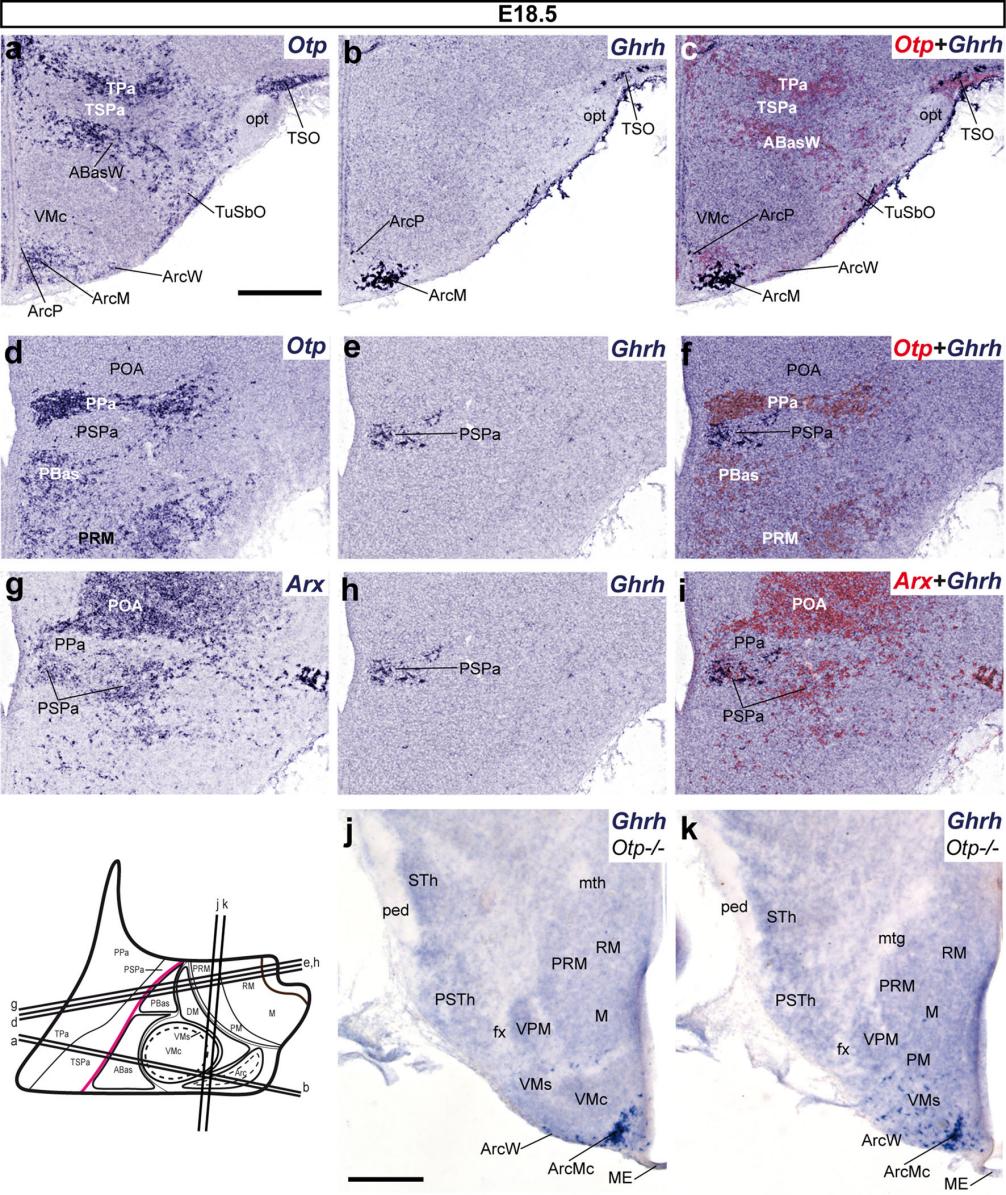
*Ghrh*+ cells mapped at E18.5. **a**–**c**, **d**–**i** Alternate transverse sections at two different rostrocaudal levels, correlating the expression patterns of indicated reference markers (*Otp, Arx*) with the presence of *Ghrh*+ cells. Corresponding digital overlaps of *pseudocolored* reference markers and *Ghrh* signal are marked with a *plus* symbol. **j**, **k** Horizontal sections at two different dorsoventral levels of *Otp*-null E18.5 embryos showing basal *Ghrh*+ cells largely restricted to the Arc tuberal area within THy (ArcM, ArcW), but extending as well into the nearby ventral shell of the VM nucleus (VMs). Transverse and horizontal section planes are shown in the adjacent schematic insert. *mth* mamillothalamic tract, *ped* cerebral peduncle,*STh* subthalamic nucleus, *PSTh* parasubthalamic nucleus, *VPM* ventral premamillary nucleus. *Scale* *bar* 300 μm

Data found at the Allen Developing Mouse Brain Atlas suggest that the *Ghrh* cell distribution is nearly definitive from E18.5 onwards (no significant change compared to P28). At E18.5, the alar plate was practically empty of *Ghrh*+ cells, with exception of remnants at the LPa and CPa (PHy), TSO and LChS (Figs. 11a–f, 12a–c) and the caudal part of PSPa (PHy) (Figs. 11g–l; 12d–i); *Ghrh*+ neurons continued to be present in restricted subareas of the basal hypothalamus, as found at E15.5 (Figs. 11; 12a–c). Specifically, *Ghrh*+ cells were found within the basal THy, at the TuSbO (Fig. 11a–f), the ventral part of VMs, and the acroterminal Arc. Interestingly, there is a new large population at the Arc medial part (an acroterminal domain; see Puelles et al. 2012), specifically restricted to its core portion (ArcM; Figs. 11g–r, 12a–c), while fewer cells were present at the ArcW, similarly as at E15.5. Within the basal PHy we observed dispersed *Ghrh*+ cells in the PBas area, in ventral continuity with similar cells in the DMsP (Fig. 11m–r).

We also mapped *Ghrh*+ cells on E18.5 mice embryos homozygous for *Otp* deletion, aiming to test the dependency of the diverse *Ghrh*+ cells groups on an *Otp*+ background (recapitulating the study of Acampora et al. 1999). It turns out that these animals show absolutely no labeled alar population (neither telencephalic nor hypothalamic; not shown), and no basal population either within PHy (PBas or DMsP; Fig. 12j, k). In contrast, the basal THy displays a normal tuberal population of *Ghrh*+ cells at the ArcM core, with less numerous cells dispersed laterally within the ArcW area and caudally under the VM nucleus (ventral VMs); no such cells were found caudal to the IHB (Fig. 12j, k). Interestingly, the TuSbO appears devoid of *Ghrh*+ cells, confirming the assumption that these cells normally originate in alar *Otp* + THy territory.

## Discussion

In the past, developmental studies of hypothalamic cell populations (usually identified as nuclei) were performed within the traditional columnar model of forebrain subdivisions (Kuhlenbeck 1973; Altman and Bayer 1986; Swanson 1992, 2003). In order to pinpoint particular cell groups, experimental autoradiographic identification of cell birthdays was often analyzed primarily at postnatal or adult stages, establishing sets of cell-birthday data that provide a sound background for developmental analysis (Angevine 1970; Ifft 1972; Altman and Bayer 1986; Markakis and Swanson 1997). In the case of the monographic study of Altman and Bayer (1986), the observed neurogenetic patterns (gradients) led to a priori postulates on hypothetic immature progenitor sites thought to give rise to specific neuronal populations. These sites were checked subsequently by sequential-labeling for neuronal production at the predicted stages, aiming to trace these populations serially across diverse stages into the mature entities (Altman and Bayer 1986, 1988). Unfortunately, the multiplicity of neighboring progenitor areas that produce neurons simultaneously in any large brain region such as the hypothalamus was generally not examined critically enough, leading to arbitrary identification of the progenitor areas. Similarly, the identity of given immature cell populations does not result sufficiently characterized at succeeding early developmental stages exclusively by their birthdates. This double problem (on top of the assumption of an unsubstantiated columnar length axis; see criticism in Puelles et al. 2012) detracts from the scientific weight of the reported ‘corroborations’ of the assumed areal origins. The postulated progenitor sources and related neuronal migratory paths obtained following this approach essentially remained conjectural.

Clearly a means to identify more precisely both the distinct progenitor domains and the different immature neuronal populations was needed. Advances in molecular biology eventually produced first immunocytochemistry and then in situ hybridization for mRNA, apart of transgenic functional analysis of development. Accrued evidence using these modern approaches has richly fulfilled the expectations, providing numerous detailed data. The classic columnar diencephalic model of Herrick (1910), wherein the hypothalamus was contemplated as a longitudinal ventral forebrain unit, seems now insufficient. This model turned out to be inconsistent with longitudinal gene patterns, causal relationships of the diencephalon and hypothalamus with axial mesoderm (origin of dorsoventral patterns), and a set of mutated mouse phenotypes (reviewed by Puelles et al. 2012). In the present scenario, the columnar model is now surpassed by the prosomeric model, whose longitudinal axis is consistent with ventralizing signal sources of the axial mesoderm (roughly as suggested by His 1893). The prosomeric model is able to contemplate finer chemo- and genoarchitectonic subdivisions of the hypothalamus within a parsimonious theory of forebrain development (Puelles and Rubenstein 2003; Martinez et al. 2012; Puelles et al. 2012).

In the present report, we examined first whether hypothalamic *Crh*-, *Trh*-, and *Ghrh*-expressing neurons originate from distinct progenitor domains, which we recently had characterized molecularly within the updated prosomeric model (Morales-Delgado et al. 2011; Puelles et al. 2012). Even though the adult distribution of such cell types may be dispersed, ontogenetic analysis allows us to ask whether their progenitors share a molecular scenario that denotes a causal common denominator for given phenotypes. Secondly, we explored the possibility that these neurons may variously disperse radially and/or tangentially to reach their definitive locations in the adult hypothalamus, similarly as recently shown for *somatostatin*-expressing neurons (Morales-Delgado et al. 2011). To that aim, we studied the prenatal spatiotemporal distribution of *Crh*, *Trh*, and *Ghrh* hypothalamic neurons relative to gene markers involved in the anteroposterior and dorsoventral regionalization of the hypothalamus (Shimamura and Rubenstein 1997; Bertrand and Dahmane 2006; Yee et al. 2009; Shimogori et al. 2010; Puelles et al. 2012) and/or the specification of distinct neuroendocrine hypothalamic cells (Acampora et al. 1999; Michaud 2001; Caqueret et al. 2005; Blechman et al. 2007). We retained from earlier autoradiographic neurogenetic studies, the notion that neuronal production in the hypothalamus is organized in an outside–in pattern.

**Fig. 13:**
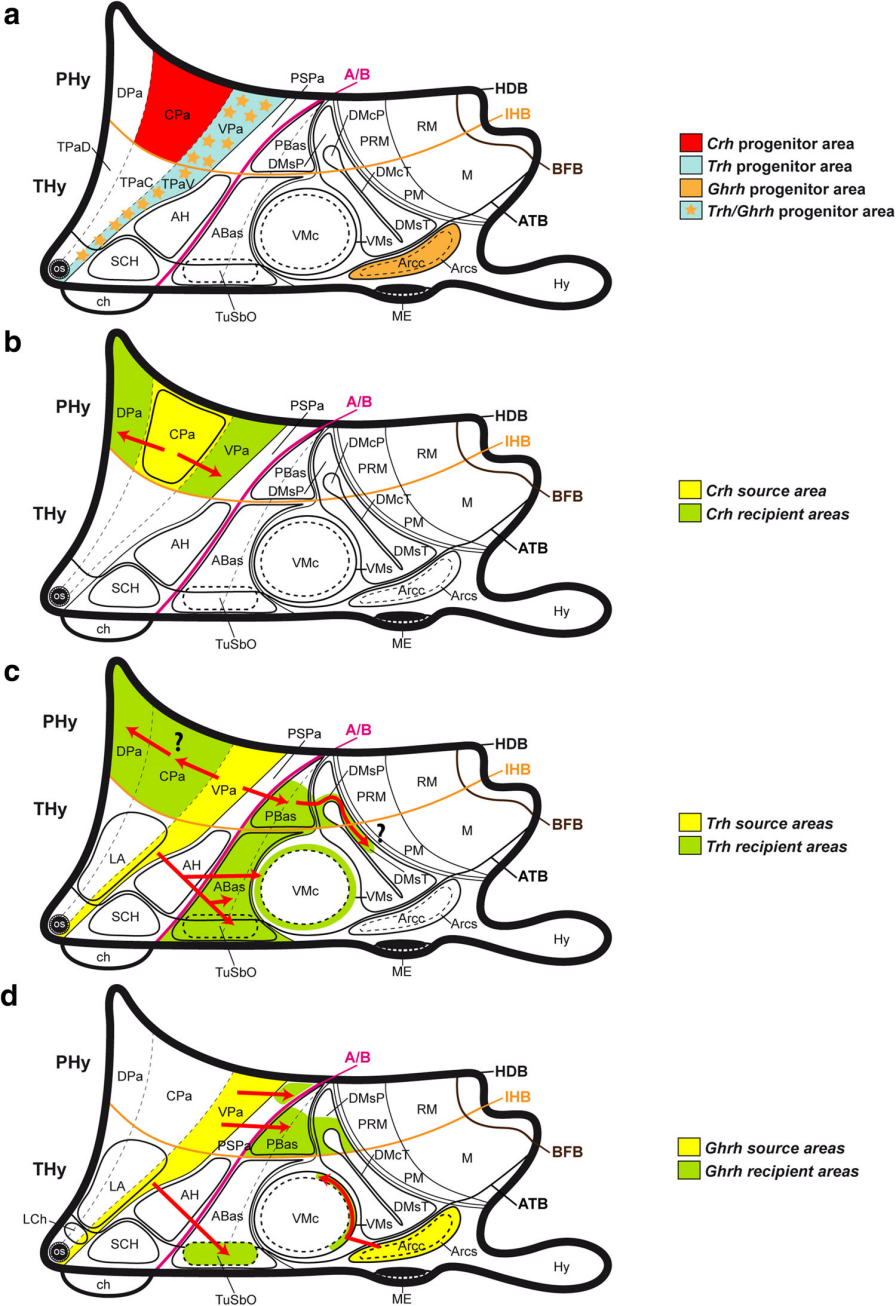
Schematic *color*-coded maps illustrating diverse prenatal progenitor areas and postulated tangential migrations routes within our model (Fig. 1c, d). **a** Diagram showing the main progenitor areas for cell populations expressing *Crh* (*red*), *Trh* (*blue*), and *Ghrh* (*orange*) during prenatal development of the mouse hypothalamus. **b**–**d** Schemata of the described *Crh*, *Trh*, and *Ghrh* source areas (*yellow*), the apparent recipient areas (*green*), and the apparent tangential migration routes (*red arrows*). Uncertain cellular migrations are indicated with a *question mark*

Relevant findings can be summarized as follows: (1) there are two discrete but longitudinally contiguous progenitor areas that produce *Trh*+ cells across alar PHy and THy (VPa and TPaV), whereas *Crh*+ cells are originated in a single more dorsal PHy alar area (CPa; Fig. 13a); *Ghrh*+ cells have a triple origin, two of them again across alar PHy and THy (VPa and TPaV areas; note that these sites are shared with *Trh*+ cells) and a separate basal one at the Arc tuberal area (no *Trh*+ cells were found here); (2) *Otp* and *Sim1* are common molecular markers associated to the alar progenitor domains mentioned above, but basal domains that also express these markers primarily (Morales-Delgado et al. 2011) do not show production of the studied phenotypes, suggesting that other determinants exclusive of the alar plate are causally relevant; (3) the *Ghrh* and *Trh* cell populations associated to the VPa and TPaV areas have a similar spatiotemporal developmental sequence, which involves partial radial migration into LPa and substantial tangential migratory dispersion ventralward, whereas *Crh* cells associated to CPa are virtually quiescent as regards tangential migration; (4) as development progresses, *Ghrh*+ and *Trh*+ cells diminished in number at the alar territories, and simultaneously increased at some basal subdivisions (PBas and DMsP in PHy; TuSbO in THy), suggesting a secondary alar–basal migratory translocation; and (5) the Arc tuberal *Ghrh*+ origin within basal THy was corroborated to be independent from any such migration by its selective persistence in the *Otp*−*/*− mouse, which lacks all the mentioned alar derivatives (Acampora et al. 1999; present results).

## Molecular profiles of postulated progenitor areas as necessary environment for cell type differentiation

Recently accrued evidence indicates that spatially restricted alar and basal neuroepithelial progenitor domains of the hypothalamus express distinct combinations of transcription factors (Puelles and Rubenstein 2003; Puelles et al. 2004, 2012; Shimogori et al. 2010). This initial regionalized state is held to imply particular differentiative potencies once neurogenesis steps in, leading to diversified mantle layer populations within prespecified boundaries (Alvarez-Bolado et al. 1995, 2012; Shimogori et al. 2010; Morales-Delgado et al. 2011; Puelles et al. 2012). However, the capacity to produce specific neuronal phenotypes is not necessarily restricted to unique progenitor domains, since alternative sites may produce neurons expressing the same differentiation marker, possibly in coexistence with some differential properties. One possible explanation of such redundancy is that some crucial molecular determinants are shared, thus explaining the partial similarity in cell fates; alternatively, in the absence of shared molecular characteristics, the global causal network of patterning genes may allow separate pathways that entail a comparable result (for instance, via different enhancers; Puelles and Ferran 2012). In our previous work on the ontogeny of *Sst*-expressing neuron populations in the mouse hypothalamus, we observed at least four independent progenitor domains (two alar and two basal) producing this cell type; all of them shared an *Otp*-expressing background (Morales-Delgado et al. 2011). Our present analysis of three other peptidergic cell populations identifies further distinct derivatives of the *Otp*-expressing background, albeit restricted to the alar plate, allowing a wider perspective of this still insufficiently understood scenario. *Otp* is known to be a key gene in the differentiation of SST-, CRH-, and TRH-producing neurons (revised by Caqueret et al. 2005), but was not held to be involved in the GHRH phenotype.

### Differentiation of neurons expressing *Crh*, *Trh*, and *Ghrh* in the alar plate of the hypothalamus

According to present results, distinct progenitor sources of *Crh*+, *Trh*+, and *Ghrh*+ cells belong to the hypothalamic alar plate (apart of a separate basal origin of a distinct subpopulation of *Ghrh*+ cells), and are subdivisions of the molecularly characteristic *Otp*+*/Sim1*+ paraventricular complex (CPa and VPa/TPaV in Fig. 13a). Due to the use of a less resolutive columnar approach, earlier autoradiographic studies of hypothalamic progenitor domains did not detect the essential distinctness of the paraventricular and subparaventricular alar areas (nor separated these efficiently from the preoptic area; e.g., Altman and Bayer 1986, 1988). Loss-of-function experiments in mice have revealed that *Otp* and *Sim1* genes, respectively, encoding homeodomain and bHLH-PAS transcriptional regulators, are required for the terminal differentiation of TRH and CRH hypothalamic parvocellular neurons within the Pa complex (Michaud et al. 1998; Acampora et al. 1999; Wang and Lufkin 2000; Goshu et al. 2004; Blechman et al. 2007; Duplan et al. 2009). In these works, the *Ghrh* lineage was not investigated within the Pa, probably because of the virtual absence of this cell type in the adult Pa complex. However, we found that the more or less dispersed GHRH cells present in the adult mouse hypothalamus (Simmons and Swanson 2009) partly originate at the ventral Pa subdomain across PHy and THy, jointly with the TRH neurons. We first identified *Ghrh*-expressing cells at the VPa at E10.5 (not previously reported), and we traced their subsequent migratory exodus into subparaventricular and basal retrotuberal areas (PSPa; PBas, DMsP). Significantly, we checked that *Otp*−*/*− mice completely lack all alar-derived and ventrally migrated *Ghrh*+ cells. These results strongly suggest that *Otp* and *Sim1* genes in the alar plate Pa domain (specifically, at the ventral subdomain of Puelles et al. 2012) may also be involved in the genetic specification of the alar GHRH cells. An independent basal origin, previously described in the rat—Rodier et al. 1990; Burgunder 1991— was also corroborated at the basal ArcM acroterminal domain of Puelles et al. (2012) and nearby tuberal areas (ArcW and ventral VMs; note these less numerous and dispersed elements appear first at E13.5, whereas the more important ArcM core group of cells differentiates between E15.5 and E18.5; this may owe to neurogenetic retardation at the acroterminal median area).

However, the activation of *Sst*, *Crh*, *Ghrh*, and *Trh* genes cannot result merely from the co-expression of *Otp* and *Sim1*. Other genes must be involved in the respective fate determinations at their specific progenitor areas. The extensive Pa mantle area is fully populated by *Otp*+ cells from very early stages of development (Simeone et al. 1994; Morales-Delgado et al. 2011) and only relatively minor subpopulations develop *Sst*+, *Crh*+, *Trh*+, and *Ghrh*+ phenotypes. Another well-known group of Pa derivatives includes the magnocellular hypophysotropic paraventricular and supraoptic neurons that express oxytocin and vasopressin (reviewed by Caqueret et al. 2005); data found at the Allen Developing Mouse Brain Atlas indicate that these markers are expressed several days later than those studied by us here (http://www.developingmouse.brain-map.org). Additional dopaminergic and glutamatergic derivatives were discussed by Puelles et al. (2012). Unfortunately, there is no general agreement about subdivisions in the Pa, partly due to conceptual and terminological differences caused by the inconciliable columnar and neuromeric models (Puelles et al. 2012). These authors proposed a new fundamental organization of the Pa area (considered to be topologically longitudinal in the prosomeric model) into peduncular (PHy) and terminal (THy) transverse (neuromeric) moieties of the hypothalamus (the major Pa nuclear complex lies in PHy, while the conventional anterior periventricular, aPV- and supraoptic nuclei, TSO, TuSbO- originate within THy; note that Altman and Bayer 1986 simply assumed that all these parts have a common origin). Both Pa parts were postulated to be further subdivided dorsoventrally (relative to the prosomeric length axis) into dorsal, central, and ventral subdomains (DPa, CPa, VPa within PHy, where this subdivision is more patent; Fig. 1b). The data reported by Michaud et al. (1998) about partial overlap of *Sim1* and *Brn2* expression in this area in wildtype and *Sim1* mutant mice embryos (in their Fig. 7; see particularly the schematic inserts) are consistent with this subdivision concept, irrespective that their morphologic interpretation was done within the columnar model (e.g., their ‘dorsal’ descriptor corresponds to our ‘caudal’ position, and their ‘caudal’ positions are ‘ventral’ in our model). *Brn2* signal apparently predominates in the ventral subdomains where *Trh*+ and *Ghrh*+ cells arise. Note that we believe some *Sim1* expression, even if weaker, also obtains there; in fact, initially, *Brn2* expression is restricted to the VPa (Goshu et al. 2004; their Fig. 4c), but extends dorsally into CPa at E13.5 (see Allen Developing Mouse Brain Atlas). *Sim1* expression is not accompanied by *Brn2* in the dorsal subdomains, but both markers overlap significantly at the central ones (Michaud et al. 1998; their Fig. 7). Accordingly, it is possible that a relatively weaker (or earlier) *Brn2* ventral signal within Pa is associated to differential early ventral production of the *Trh* and *Ghrh* parvocellular phenotypes, whereas parallel strong levels of expression of both markers may correlate with *Crh* cells formed at the CPa. The transcription factor *Brn2* was shown to be required for the survival and maintenance of differentiated CRH neurons at the CPa subdomain of the paraventricular nucleus (Nakai et al. 1995; Schonemann et al. 1995; Caqueret et al. 2005). Other peculiarities can be expected at the DPa, where *Brn2* seems absent (for instance, dopaminergic neurons seem concentrated there; Puelles et al. 2012; their Fig. 8.25). Moreover, Michaud et al. (1998) observed progressive downregulation of *Brn2* at the PHy (*Sim1*-dependent maintenance) in the *Sim1* mutant, but the expression was respected at the THy. These data support our neuromeric interpretation of their results, which notably cannot be explained within the columnar model.

Such regional differences may be complemented by additional molecular determinants underpinning the positional restriction of *Crh*+ cells to the CPa subdomain, and of alar *Trh*+ and *Ghrh*+ cells to the TPaV and VPa (Fig. 13a). A possible additional factor to be considered in this context is *Sim2*, a paralog of *Sim1* that is activated downstream of it (Wang and Lufkin 2000; Goshu et al. 2004). The *Sim2* mouse mutant (*Sim2*−/−) has a reduced number of TRH and SST neurons in the paraventricular and anterior periventricular nuclei (Goshu et al. 2004), thus suggesting selective effects within the ventral Pa subdomains, though it is not evident that *Sim2* is expressed selectively or differentially at this location (Fan et al. 1996). No data are available for GHRH neurons in this mutant. More detailed analysis of the *Sim2* expression pattern over time is therefore needed.

The *Dickkopf 3* (*Dkk3*) gene, a member of the *Dickkopf* family of *Wnt* modulators, which is otherwise involved in survival, migration and morphologic differentiation of neuronal retinal cells (Iida et al. 2011), also may be related to the differentiation of CRH neurons at the CPa, since it is selectively and abundantly expressed there from E11.5 onwards (see Allen Developing Mouse Brain Atlas).

One point that remains obscure is what causes parallel production of *Trh* and *Ghrh* cells within the ventral subdomains of the hypothalamic paraventricular area. Both cell types first appear at E10.5, and represent a small proportion of the cells present in the immature mantle layer (all *Otp*+). One possibility is that future studies may discover even earlier microareal molecular differences within the Pa subdomain considered here, which may underlie a differential pathway choice, with subsequent cell mixing in the mantle layer. Recently, Guerra-Crespo et al. (2011) reported about specific transcriptome elements associated to the *Trh* phenotype. We checked the *Klf4*, *Klf10*, and *Atf3* genes underlined in that study in the Allen Developing Mouse Brain Atlas, but did not find significative expression recorded for any of them at E11.5 or E13.5. Another explanatory possibility to be considered is that mutual intercellular signaling analogous to Delta/Notch signaling may contribute to these early differentiative steps, so that, e.g., differentiating *Trh* cells cause surrounding postmitotic neurons to become *Ghrh* cells, or vice versa.

Finally, it is also of interest that the *Otp*+ ventral Pa progenitor sources of alar *Trh* and *Ghrh* cells are selectively recipient of a population of tangentially migrating *Nkx2.2*+ cells (Puelles 2000; Puelles et al. 2012), an independent process that corroborates the local existence of idiosyncratic molecular properties. Further differential behavior occurring at this ventral alar site is represented by the radial dissociation of the LPa nucleus within VPa (nothing similar occurs at the CPa and DPa subareas). It is presently unclear whether the neighborhood of VPa/TPaV with the subjacent *Arx*+*/Dlx5*+ subparaventricular domain (the ventralmost alar domain across PHy and THy; Bulfone et al. 1993; Puelles and Rubenstein 1993; Puelles et al. 2004, 2012; Shimogori et al. 2010) is somehow causally related to the discussed differential histogenetic properties within the Pa complex.

### Separate origin of neurons expressing *Ghrh* in the basal plate of the hypothalamus

In our material *Ghrh*+ cells start to populate the arcuate area at E13.5, in the form of dispersed cells, but a denser bilateral population appears at the ArcM core after E15.5 (Figs. 9; 12j, k; 13a). Immunocytochemical and in situ hybridization studies done previously in the rat already identified these cells; it was held that the arcuate area was the sole origin of this cell type (Rodier et al. 1990; Burgunder 1991). Data in the literature accordingly linked the neuronal *Ghrh* phenotype to the function of genes expressed primarily in the tuberal basal plate, specifically at the Arc. During early development, ventralizing SHH signals from the axial mesoderm induce expression of *Nkx2.1* in the overlying basal hypothalamus. This promotes differential molecular development of this region at later stages (Ericson et al. 1995; Kimura et al. 1996; Pabst et al. 2000; Marin et al. 2002; Puelles et al. 2004, 2012; Shimogori et al. 2010). Subsequent expression of the homeobox genes *Hmx2, Hmx3,* and *Mash1* in the tuberal area (reputedly also at the Arc nucleus, at its acroterminal end) has been related to activation and maintenance of *Gsh1* expression and subsequent downstream generation of *Ghrh*-expressing neurons (Wang et al. 2004; Caqueret et al. 2005; McNay et al. 2006; Jo and Chua 2009; Szarek et al. 2010). Notably, *Hmx2/Hmx3* null mice exhibit absence of *Gsh1* expression and of GHRH-immunoreactive cells at the Arc (Wang et al. 2004). Expression of *Gsh1* at the Arc is held to directly regulate *Ghrh* expression by interaction with its promoter (Li et al. 1996).

This evidence implies a local basal origin of *Ghrh*+ cells that is separate from the alar one discussed above. The former is unrelated to the expression of *Otp/Sim,* as is clearly indicated by the observation that *Otp*-null mice have a normal Arc *Ghrh*+ population (plus caudally dispersed cells in the ventral shell area of the VM nucleus; Acampora et al. 1999; their Figs. 3K, K’; 4L, L’), but lack completely the alar-derived subparaventricular and retrotuberal elements (present results; Figs. 12j, k, 13d). Complementary data shown by Wang et al. (2004) indicate that in *Hmx2/Hmx3* null mice GHRH-immunoreactive cells are absent both at the Arc and VM shell, but are apparently present (after migration) within the retrotuberal area; we think these retrotuberal elements were wrongly identified as ‘VM cells’ by these authors (Wang et al. 2004; arrowhead in their Fig. 4Bb). These data imply a wholly different causal pathway leading separately to the *Ghrh* phenotype, lacking upstream *Otp/Sim1* activity, but requiring *Hmx2/Hmx3* and *Gsh1* functions.

## Postulated migratory routes into other hypothalamic subdomains

Description and causal explanation of any radial and tangential cell migrations in the developing mantle layer of the hypothalamus are necessary steps for unraveling its structural complexity in the adult. Various neuronal tangential migrations occur strictly inside the hypothalamus (Skidmore et al. 2008; Alvarez-Bolado et al. 2000; Puelles et al. 2012), or penetrate the hypothalamus from regions outside it (Schwanzel-Fukuda and Pfaff 1989; Wray et al. 1989; Zhao et al. 2008). For instance, our previous work suggested an intrahypothalamic tangential dorsoventral migration of *Sst* cells within the basal plate (Morales-Delgado et al. 2011). Indeed, a subset of *Sst* cells produced at the acroterminal ABas domain disperses ventrally into the shell of the VM and the Arc nucleus. Unfortunately, earlier studies focused on the ontogeny of peptidergic neurons in the rodent hypothalamus (e.g., those reviewed by Markakis 2002) did not take into account at all the possibility of tangential migrations, giving the impression of highly heterogeneous origins of multiple cell types.

As far as we know, no explicit record exists of tangentially migrating *Trh* and *Ghrh* cells in the vertebrate hypothalamus, in spite of the ventralward dispersion of GHRH cells that can be deduced from a previous descriptive study in the rat (Rodier et al. 1990). Notably, the autoradiographic analysis of Altman and Bayer (1986, 1988) did not detect the dorsoventral alar–basal translocations observed in our material. Our interpretations with respect to tangential migrations are based on the temporal sequence and relative density of *Trh*+ and *Ghrh*+ cells successively present at locations that are differentially characterized in molecular terms (alar vs. basal markers). We hold that the alternative possibility that these time-series of data can be explained by a sequential process of gene expression regulation (the marker being turned off in some cells, and simultaneously turned on in different but neighboring cells, repeatedly) is quite low, apart that such an interpretation would be highly unparsimonious, since it greatly expands the number of causal molecular mechanisms needed for a full explanation. Nevertheless, the suggestions about migrations we discuss below need to be interpreted with caution, and may require additional corroborative studies.

### Migration of Crh cells into neighboring alar paraventricular subdivisions

As stated above, *Crh* cells barely migrate into other hypothalamic subareas during mouse embryonic development. *Crh*+ cells were first observed at the CPa at E13.5, and they increased in number by E15.5, still forming a rather dense subpopulation. At E18.5 they became slightly less compact, so that some *Crh*+ cells were found as well in both the dorsal and ventral subdivisions of the Pa complex (DPa, VPa, LPa; Fig. 2q). This suggests some retarded and rather limited tangential displacement of *Crh* cells from CPa into DPa and VPa (Fig. 13b).

### Apparent displacement of Trh cells within the alar paraventricular area

The original population of *Trh*+ cells observed at the VPa surprisingly seems to diminish by E13.5. Previously, a small group of such cells appears at the CPa (Fig. 3j). At E15.5 and E18.5, there remain practically no *Trh*+ cells at the VPa, and a sizeable population is found instead at the CPa and DPa (Figs. 5l, o, r, 7i, l). This sequence may be understood as evidence of dorsalward tangential migration of a subgroup of VPa *Trh*+ cells into CPa and DPa (question mark; Fig. 13c). Alternatively, the CPa and DPa cells may have been generated ex novo at these sites, bespeaking only of a retarded neurogenetic pattern with respect to this phenotype, irrespective of a parallel depletion of the VPa in a different direction (see below for apparent displacement into the underlying PSPa and basal plate). However, this would require less parsimonious sets of causal explanations about the origin of such cells.

### Migration of alar *Trh*- and *Ghrh*-positive cells into basal hypothalamic sites

A detailed comparison of *Ghrh* and *Trh* expression patterns at relevant developmental stages indicates that cell populations showing these two markers increase at several basal plate sites, and appear as well transiently at intermediate alar positions (the subparaventricular area), while diminishing at the presumptive original paraventricular progenitor domain. This pattern strongly suggests that members of both populations migrate ventralward from their alar source into the basal plate (Fig. 13c, d).

The sequence evolves as follows: between E10.5 and E13.5, a part of the *Trh*+/*Ghrh*+ cells produced at the VPa first become displaced ventrally into the subjacent alar PSPa domain, and subsequently diminish there and appear within the underlying basal domain, PBas. The latter is a retrotuberal subdomain characterized molecularly by selective expression of *Lhx9* at embryonic stages (Shimogori et al. 2010) and weak or less apparent *Nkx2.1* signal (otherwise widespread in the hypothalamic basal plate; this signal possibly is concentrated here at deep periventricular levels); PBas displays some dispersed *Otp*+ or *Dlx5*+ cells, which may be also migrated, at least in part (Morales-Delgado et al. 2011; Puelles et al. 2012). The PBas corresponds to the area previously identified as posterior entopeduncular area (PEP in Bulfone et al. 1993; Puelles and Rubenstein 2003; Puelles et al. 2004). From E15.5 onwards, *Trh*+ and *Ghrh*+ cells diminish or disappear at the PSPa, persist at the PBas, and newly appear within the underlying peduncular DM shell domain (DMsP). Similar paraventricular *Trh*+ cells born at the THy transiently cross dorsoventrally the TSPa (Fig. 4n) and aggregate below at the ABas and TuSbO nuclei (which is largely formed by migrated alar *Otp*+ cells). A small subgroup of *Ghrh*+ cells apparently accompanies them. These data suggest that alar peduncular *Trh*+ and *Ghrh*+ cells (the major alar population) migrate first dorsoventrally into the peduncular basal plate (retrotuberal area), where they are still visible in the adult. Similarly, the smaller alar TPaV populations of *Trh*+ cells and *Ghrh*+ cells also diminish in number in favor of basal ones (ABas; TuSbO; note other elements may migrate dorsalward into the preoptic area), suggesting a smaller but parallel alar–basal translocation within THy (Fig. 13c).

Apart of the cells apparently invading the basal plate, a group of *Ghrh* cells remains visible within the caudal part of PSPa (Fig. 9l), while such cells essentially disappear from the overlying VPa origin. In contrast, some *Trh*+ cells continue present to some extent within Pa (see section above), but subparaventricular locations for this cell type were transient, and they largely incorporate into the basal plate. Within our migratory interpretation of these data, some *Ghrh*+ cells would migrate selectively from VPa into PSPa, becoming there stabilized, independently of those entering the basal plate (Fig. 13d). No *Trh*+ cell imitates them in this regard.

The classic VM and DM nuclei are, respectively, the major components of the intermediate Tu/RTu areas (Fig. 1c; Puelles et al. 2012). The VM appears restricted to the upper part of TuI in adults, whereas the DM is currently identified as a longitudinal entity that begins caudally within RTuI (peduncular DM) and extends into the TuI, ventrally to the VM (terminal DM); the latter part ends caudally to the acroterminal Arc complex (Puelles et al. 2012; their Fig. 8.31). Both VM and DM are heterogeneous entities composed of different cell types whose identity and spatial distribution have not been fully resolved yet. Various chemo- and genoarchitectonic mappings suggest in both cases a fundamental organization into compact *core* portions and surrounding dispersed *shell* domains (Milhouse 1973; Chou et al. 2001; Choi et al. 2005; Segal et al. 2005; McClellan et al. 2006; Lee et al. 2012; Puelles et al. 2012). The latter authors noted that the VM and DM core domains are nearly completely excitatory (glutamatergic), whereas their shell regions, VMs, DMs, contain more dispersed inhibitory GABAergic neurons intermixed with glutamatergic ones (Puelles et al. 2012; their Figs. 8.17–8.24). Whereas the peduncular and terminal DMs populations seem to develop locally and express *Dlx5* (intermediate retrotuberal and tuberal areas, respectively), the peduncular and terminal DM core populations probably are elements tangentially migrated from more dorsal basal origins; (Puelles et al. 2012; their Figs. 8.15; 8.26L–N). In contrast, the whole VM complex (core and shell parts) translocates dorsoventrally between E13.5 and E15.5 from a dorsal tuberal origin close to the *Nkx2.2*+ alar–basal boundary (Puelles et al. 2012; their Fig. 8.26). In the rat, several developmental studies have previously described the presence of *Trh*-expressing neurons within the DM at E15–E16 (Burgunder and Taylor 1989) and TRH-immunoreactive neurons were reported in the VM at E15.5 (Okamura et al. 1991). Burgunder (1991) observed dispersed *Ghrh*-expressing cells in what we interprete as the VMs at E17, though he did not specify a core versus shell position. Markakis and Swanson (1997) also reported parvicellular neuroendocrine GHRH and TRH neurons within the VM and DM nuclei of the adult rat; these authors apparently assumed these neurons had originated there, but we propose they should be interpreted as migrated cells after the present study of the molecular ontogenetic scenario. It is of interest that the BrdU-labeling data reported in that study suggest that relatively late-generated cohorts (after E14) are selectively those that colonize the basal plate, whereas cells born earlier tend to remain in the alar plate.

Taken together, these data suggest that the majority of the presently postulated tangential hypothalamic migrations take place dorsoventrally within either the peduncular (caudal) or terminal (rostral) hypothalamic prosomeric regions where the different cell cohorts arose. Quantitatively the phenomenon is more important within PHy. We have not found so far any evidence of neurons moving from PHy into THy, or vice versa. Puelles et al. (2012) already underlined the majoritary course of dorsoventral hypothalamic tracts through the PHy. These include the *fornix*, the *medial* *forebrain* *bundle* (with some 50 individual components; Nieuwenhuys et al. 1982) and the compact *lateral* *forebrain* *bundle* itself (widely known as the cerebral peduncle). The abundance of dorsoventrally oriented fibers within the PHy, which seem quite precocious in their growth (Tello 1934), may be directly related as a physical background (neurotropic contact-guidance) to the prevalence of dorsoventral migrations (or ventrodorsal ones, as in the case of the subthalamic nucleus) in this part of the hypothalamus. Further research is needed to assess the potential importance of tissue-fixed epitopes, soluble attractive/repulsive molecular signals, or even merely permissive mechanical circumstances affecting these cellular displacements.

In summary, our study has characterized in detail an unexpectedly diversified ontogenetic profile for hypothalamic *Crh*-, *Trh*-, and *Ghrh*-expressing neurons. We provided some clues on their specific progenitor areas and the molecular backgrounds associated to them. Even more so than in our prior work that was focused on *Sst* cells (Morales-Delgado et al. 2011), some of these peptidergic cell populations (e.g., alar-derived *Trh*+ cells) show relatively simple and localized origins, and later distribute amply but selectively via radial and tangential migrations to other parts of the developing hypothalamus (alar and basal). This produces their respective adult distribution in distinct basal plate subdomains, with partial or nearly total depletion of the original alar sites where they initially emerged. Previously unknown migratory routes were highlighted in this approach. *Ghrh* cells clearly arise independently at separate alar and basal areas, similarly as previously found for *Sst* cells (Morales-Delgado et al. 2011), though in this case the respective genetic background is quite different, possibly bespeaking of a role for different enhancers upstream of the studied differentiation marker. Like *Sst* and *Trh* cells, the alar *Ghrh* cells also migrate preferentially ventralward, with minor dorsalward translocations. In contrast, *Crh* cells are exceptionally quiescent, remaining centered near their origin at the central paraventricular area. It can be concluded definitively that there is no single pattern for the development of all peptidergic cell types in the hypothalamus. This forces a specific analysis for each population.

## Abbreviations

3v: Third ventricle
A/B: Alar–basal boundary
ABas: Acroterminal anterobasal nucleus
ABasM: Median acroterminal ABas
ABasW: Wings of ABas
AH: Anterior hypothalamic nucleus
AHy: Adenohypophysis
Arc: Arcuate nucleus
Arcc: Core portion of the Arc
ArcM: Median acroterminal arcuate nucleus
ArcMc: Core portion of median acroterminal arcuate nucleus
ArcP: Periventricular stratum of Arc
Arcs: Superficial stratum of the Arc
ArcW: Wings of Arc
Arx: Aristaless gene
ATB: Acroterminal border
BFB: Basal-floor boundary
ch: Optic chiasm
CPa: Central portion of paraventricular area
Crh: Corticotrophin-releasing hormone gene
Dg: Subpallial diagonal area
Dienc: Diencephalon
Dlx5: Distal-less 5 gene
DM: Dorsomedial hypothalamic nucleus
DMcP: Core portion of the peduncular DM
DMcT: Core portion of the terminal DM
DMsP: Shell portion of the peduncular DM
DMsT: Shell portion of the terminal DM
DPa: Dorsal portion of paraventricular area
EPV: Ventral entopeduncular nucleus
ESO: Episupraoptic nucleus
fx: Fornix tract
Ghrh: Growth hormone-releasing hormone gene
Gsh1: Gamma-glutamylcysteine synthethase 1 gene
HDB: Hypothalamo-diencephalic boundary
Hy: Hypophysis
icms: Infracapsular migration
IHB: Intrahypothalamic boundary
LA: Lateroanterior hypothalamic nucleus
LCh: Laterochiasmatic nucleus
LChS: Superficial laterochiasmatic nucleus
LPa: Lateral paraventricular nucleus
M: Mamillary body
ME: Median eminence
Mes: Mesencephalon
MGE: Medial ganglionic eminence
ML: Lateral mamillary nucleus
MML: Lateral mamillary area
mtg: Mamillotegmental tract
NHy: Neurohypophysis
Nkx2.1: Nk2 homeobox 1 gene
Opt: Optic tract
Os: Optic stalk
Otp: Orthopedia gene
p1: Prosomere 1
p2: Prosomere 2
p3: Prosomere 3
Pa: Paraventricular complex
PBas: Posterobasal nucleus
PFx: Perifornical nucleus
PHy: Peduncular hypothalamus
PM: Perimamillary area
POA: Preoptic area
POH: Preopto-hypothalamic transition area of the hypothalamic ventricular organ
PPa: Peduncular domain of Pa
PRM: Periretromamillary area
PRML: Superficial periretromamillary area
PSPa lim: PSPa borderline
PSPa: Peduncular domain of SPa
PTh: Prethalamus
PThE: Prethalamic eminence
Rhomb: Rombencephalon
RM: Retromamillary region
RML: Lateral retromamillary region
RTu: Retrotuberal area
RTuD: Dorsal retrotuberal domain
RTuI: Intermediate retrotuberal domain
RTuV: Ventral retrotuberal domain
SCH: Suprachiasmatic nucleus
scms: Supracapsular migration
Sim1: Single-minded homolog 1
SPa: Subparaventricular area
THy: Terminal hypothalamus
TPa: Terminal domain of Pa
TPaC: Central portion of TPa
TPaD: Dorsal portion of TPa
TPaV: Ventral portion of TPa
Trh: Thyrotrophin-releasing hormone gene
TSO: Terminal supraoptic nucleus
TSPa lim: TSPa borderline
TSPa: Terminal domain of SPa
Tu: Tuberal region
TuD: Dorsal tuberal domain
TuI: Intermediate tuberal domain
TuSbO: Suboptic nucleus
TuV: Ventral tuberal domain
VM: Ventromedial hypothalamic nucleus
VMc: Core portion of the VM
VMs: Shell portion of the VM
VPa: Ventral portion of paraventricular area
ZIR: Prethalamic zona incerta
